# ﻿Comparative karyotype analysis of eight Cucurbitaceae crops using fluorochrome banding and 45S rDNA-FISH

**DOI:** 10.3897/compcytogen.17.99236

**Published:** 2023-02-09

**Authors:** Chao-Wen She, Xiang-Hui Jiang, Chun-Ping He

**Affiliations:** 1 Key Laboratory of Research and Utilization of Ethnomedicinal Plant Resources of Hunan Province, Huaihua University, Huaihua, Hunan, 418008, China Huaihua University Huaihua China; 2 College of Life Sciences and Chemistry, Hunan University of Technology, Zhuzhou, Hunan, 412007, China Hunan University of Technology Zhuzhou China

**Keywords:** Cucurbitaceae, cytotaxonomy, fluorescence *in situ* hybridization, fluorochrome banding, karyotype, karyotype asymmetry, ribosomal RNA genes (rDNA)

## Abstract

To have an insight into the karyotype variation of eight Cucurbitaceae crops including *Cucumissativus* Linnaeus, 1753, *Cucumismelo* Linnaeus, 1753, *Citrulluslanatus* (Thunberg, 1794) Matsumura et Nakai, 1916, *Benincasahispida* (Thunberg, 1784) Cogniaux, 1881, *Momordicacharantia* Linnaeus, 1753, *Luffacylindrica* (Linnaeus, 1753) Roemer, 1846, Lagenariasicerariavar.hispida (Thunberg, 1783) Hara, 1948 and *Cucurbitamoschata* Duchesne ex Poiret, 1819, well morphologically differentiated mitotic metaphase chromosomes were prepared using the enzymatic maceration and flame-drying method, and the chromosomal distribution of heterochromatin and 18S-5.8S-26S rRNA genes (45S rDNA) was investigated using sequential combined PI and DAPI (CPD) staining and fluorescence *in situ* hybridization (FISH) with 45S rDNA probe. Detailed karyotypes were established using the dataset of chromosome measurements, fluorochrome bands and rDNA FISH signals. Four karyotype asymmetry indices, CV_CI_, CV_CL_, M_CA_ and Stebbins’ category, were measured to elucidate the karyological relationships among species. All the species studied had symmetrical karyotypes composed of metacentric and submetacentric or only metacentric chromosomes, but their karyotype structure can be discriminated by the scatter plot of M_CA_ vs. CV_CL_. The karyological relationships among these species revealed by PCoA based on *x*, 2*n*, TCL, M_CA_, CV_CL_ and CV_CI_ was basically in agreement with the phylogenetic relationships revealed by DNA sequences. CPD staining revealed all 45S rDNA sites in all species, (peri)centromeric GC-rich heterochromatin in *C.sativus*, *C.melo*, *C.lanatus*, *M.charantia* and *L.cylindrica*, terminal GC-rich heterochromatin in *C.sativus*. DAPI counterstaining after FISH revealed pericentromeric DAPI^+^ heterochromatin in *C.moschata*. rDNA FISH detected two 45S loci in five species and five 45S loci in three species. Among these 45S loci, most were located at the terminals of chromosome arms, and a few in the proximal regions. In *C.sativus*, individual chromosomes can be precisely distinguished by the CPD band and 45S rDNA signal patterns, providing an easy method for chromosome identification of cucumber. The genome differentiation among these species was discussed in terms of genome size, heterochromatin, 45S rDNA site, and karyotype asymmetry based on the data of this study and previous reports.

## ﻿Introduction

Cucurbitaceae, which is among the economically most important plant families, consists of about 123 genera with over 800 species distributed most in tropical and subtropical areas and very rare in temperate regions (Jeffrey 2005). Cucurbitaceous species (cucurbits) have a large range of fruit characteristics, and are cultivated worldwide in a variety of environmental conditions (Bisognin 2002). Among the cultivars of this family, cucumber (*Cucumissativus* Linnaeus, 1753), melon (*Cucumismelo* Linnaeus, 1753), watermelon (*Citrulluslanatus* (Thunberg, 1794) Matsumura et Naka, 1916), wax gourd (*Benincasahispida* (Thunberg, 1784) Cogniaux, 1881), bitter gourd (*Momordicacharantia* Linnaeus, 1753), sponge gourd (*Luffacylindrica* (Linnaeus, 1753) Roemer, 1846), bottle gourd (*Lagenariasiceraria* (Molina) Standley, 1930), squash and pumpkin (*Cucurbita* Linnaeus, 1753), all of which belong to subfamily Cucurbitoideae (Jeffrey 2005), are grown as vegetable crops with global or local economic importance, providing human with edible and medicinal fruits (Bisognin 2002).

In higher plants, karyotype analysis has been used to characterize the genome at chromosome level, to elucidate cytotaxonomic relationships among taxa, to reveal the genetic aberrations, to understand the trends in chromosome evolution, to integrate genetic and physical maps (Moscone et al. 1999; Peruzzi et al. 2009, 2017; Han et al. 2011; Guerra 2012; Siljak-Yakovlev and Peruzzi 2012; She and Jiang 2015; She et al. 2015, 2017, 2020; Astuti et al. 2017; Kadluczka and Grzebelus 2021). In general, a description of the karyotype includes the chromosome number, the absolute and relative length of chromosomes, the position of primary and secondary constrictions, the distribution of heterochromatic segments, the number and position of rDNA sites and other DNA sequences, and the degree of asymmetry (Li and Chen 1985; Levin 2002; She and Jiang 2015; She et al. 2015, 2017, 2020). Among the karyotypic parameters, the karyotype asymmetry, which is determined by the variation in chromosome length (interchromosomal asymmetry) and the variation in centromere position (intrachromosomal asymmetry) in a chromosome complement, is an important karyotype character reflecting the general morphology of chromosomes, and is thus widely used in plant cytotaxonomy (Stebbins 1971; Paszko 2006; Peruzzi et al. 2009, 2017; Peruzzi and Eroglu 2013; Astuti et al. 2017; Dehery et al. 2020; Kadluczka and Grzebelus 2021).

In most cases, karyotyping is hampered by the lack of chromosome markers, which limits the identification of individual chromosomes. To overcome this obstacle, Giemsa and fluorochrome banding techniques as well as fluorescence *in situ* hybridization (FISH) technologies were successively applied in plant chromosome analysis. Double fluorochrome staining, such as CMA (chromomycin A3)/ DAPI (4,6-diamidino-2-phenylindole) staining, and PI (propidium iodide)/ DAPI staining (called CPD staining) were employed to reveal simultaneously GC-rich and AT-rich heterochromatic regions on chromosomes (Schweizer 1976; She et al. 2006, 2015). FISH with repetitive DNA sequences as well as large-insert genomic DNA clones on mitotic metaphase or pachytene chromosomes can generate specific signal pattern in a plant species (Moscone et al. 1999; Hasterok et al. 2001; Koo et al. 2010; Liu et al. 2010; She and Jiang 2015; She et al. 2015, 2017, 2020). Both fluorochrome bands and FISH signals are effective markers for chromosome identification. Using the combined data of chromosome measurements, fluorochrome bands and FISH signals, we can construct detailed molecular cytogenetic karyotype of a plant species that displays morphological characteristics of chromosomes, distribution of heterochromatin and locations of DNA sequences (de Moraes et al. 2007; She and Jiang 2015; She et al. 2015, 2017, 2020). Comparison of karyotypes taking advantage of molecular cytogenetics can provide valuable information on the phylogenetic relationships and chromosome evolution among related species (Moscone et al. 1999; de Moraes et al. 2007; Weiss-Schneeweiss et al. 2008; Siljak-Yakovlev and Peruzzi 2012; She et al. 2015, 2017, 2020).

Cytogenetic studies of cucurbits started in 1920s. Earlier cytogenetic studies restricted to chromosome counting to determine the basic chromosome numbers of this family, as well as karyomorphological descriptions of some species, mainly focused on *Cucumis* Linnaeus, 1753 and *Citrullus* Schrader, 1836 (Bhaduri and Bose 1947; Trivedi and Roy 1970; Singh and Roy 1974; Turkov et al. 1975; Dane and Tsuchiya 1976; Ramachandran and Seshadri 1986; Li 1989; Beevy and Kuriachan 1996). The family was found to have several basic numbers such as *x* = 7, 8, 9, 10, 11, 12, 13, 14, 15, 16, and 20, of which *x* = 11 is the ancestral number (Carta et al. 2020). Cytogenetic observations also revealed that, except for a species of *Benincasa* Savi, 1818, the mitotic chromosomes of all other cucurbits investigated so far were rather small in size and similar in morphology, resulting in the difficulty of chromosome identification using conventional cytological procedures (Bhaduri and Bose 1947; Trivedi and Roy 1970; Singh and Roy 1974; Li 1989). C-banding technique and CMA/DAPI staining were employed for the characterization of cucumber chromosomes, revealing that individual chromosomes could be distinguished by the C- or fluorochrome banding patterns (Chen et al. 1998; Hoshi et al. 1998; Plader et al. 1998). However, the C- and fluorochrome banding techniques have rarely been successfully applied in other cucurbits till now. In recent two decades, FISH technologies have been employed in the chromosome analysis of more than 60 Cucurbitaceous species. FISH with repetitive DNA sequences, fosmid or BAC (artificial bacterial chromosome) clones, and bulked oligonucleotides probes on mitotic metaphase or pachytene chromosomes were used for karyotyping (Koo et al. 2002, 2005; Li et al. 2007; Xu et al. 2007; Han et al. 2008; Liu et al. 2010; Waminal et al. 2011; Waminal and Kim 2012, 2015; Zhang et al. 2015a, 2015b; Pellerin et al. 2018a, 2018b; Xie et al. 2019b), comparative cytogenetic analysis (Han et al. 2009; Koo et al. 2010; Zhao et al. 2011; Yagi et al. 2015; Zhang et al. 2015a; Li et al. 2016; Zhang et al. 2016), construction of cytogenetic map (Ren et al. 2009, 2012; Han et al. 2011; Sun et al. 2013), and chromosome-specific painting (Han et al. 2015). FISH experiments with 45S rDNA alone or both 5S and 45S rDNA as probes have been performed in a lot of cultivated and wild cucurbits including the eight cultivated species investigated herein (Chen et al. 1999; Hoshi et al. 1999; Koo et al. 2002; Li et al. 2007; Xu et al. 2007; Han et al. 2008; Liu et al. 2010; Waminal et al. 2011; Zhao et al. 2011; Waminal and Kim 2012, 2015; Guo et al. 2013; Reddy et al. 2013; Yagi et al. 2015; Zhang et al. 2015b; Li et al. 2016; Zhang et al. 2016; Pellerin et al. 2018a, 2018b; Xie et al. 2019b). In cucumber, the FISH signals of both 45S rDNA and centromeric satellite Type III allow for unequivocal identification of all mitotic metaphase chromosomes (Han et al. 2008). Also, the 45S rDNA FISH and self-GISH signal patterns enabled individual chromosomes of cucumber to be characterized (Zhang et al. 2015b). However, in the other seven cultivated species involved in this study, the rDNA sites can only mark a minority of the chromosomes in the complement (Chen et al. 1999; Li et al. 2007; Xu et al. 2007; Waminal et al. 2011; Ren et al. 2012; Waminal and Kim 2012; Guo et al. 2013; Li et al. 2016; Reddy et al. 2013; Xie et al. 2019b). In melon, a combination of CentM, 45S rDNA, and 5S rDNA with 21 fosmids of cucumber enabled each of the 12 chromosome pairs to be identified (Liu et al. 2010). As a whole, the karyotypes of cucumber and melon have been adequately investigated using molecular cytogenetic methods, while those of the other six species involved in this study have not been well molecular-cytogenetically studied. The karyotype of cucumber has been standardized (Han et al. 2008), but the karyotype data of the other seven species were incomplete, and showed inconsistency among the previous reports (Li 1989; Li et al. 2007; Xu et al. 2007; Liu et al. 2010; Waminal et al. 2011; Waminal and Kim 2012; Guo et al. 2013). Further cytogenetic investigations are needed for establishment of detailed karyotypes of the eight Cucurbitaceae crops to elucidate the genome differentiation at chromosome-level.

In the current study, using the enzymatic maceration and flame-drying (EMF) method, well morphologically differentiated mitotic metaphase chromosomes of the eight Cucurbitaceae crops were prepared. The chromosomes were characterized by sequential CPD staining and FISH with 45S rDNA probe. Detailed karyotypes of these species were quantitatively constructed using the combined data of chromosome measurements, fluorochrome bands and 45S rDNA FISH signals. Four different karyotype asymmetry indices of each species were calculated for evaluating the karyological relationships among these species. The molecular cytogenetic karyotypic data were assessed to gain insights into the genome differentiation and evolutionary relationships among the eight species.

## ﻿Material and methods

### ﻿Plant material

The seeds of *Cucumissativus* Linnaeus, 1753, *Cucumismelo* Linnaeus, 1753, *Citrulluslanatus* (Thunberg, 1794) Matsumura et Nakai, 1916, *Benincasahispida* (Thunberg, 1784) Cogniaux, 1881, *Momordicacharantia* Linnaeus, 1753, *Luffacylindrica* (Linnaeus, 1753) Roemer, 1846, Lagenariasicerariavar.hispida (Thunberg, 1783) Hara, 1948 and *Cucurbitamoschata* Duchesne ex Poiret, 1819 were obtained from commercial seed companies in China. Cultivar accessions used in this study are described in Suppl. material 1.

### ﻿Chromosome preparation

The seeds were germinated on moist filter paper in Petri dishes at 28 °C in the dark. Actively growing root tips were excised and treated in saturated α-bromonaphthalene at 28 °C for 1.0 h, and then fixed in a freshly prepared mixture of methanol and glacial acetic acid (3:1, v/v) at 4 °C, overnight. Mitotic metaphase chromosome spreads were prepared from meristem root tip cells according to She et al. (2006). The fixed root tips (2–3 mm) were thoroughly washed in double distilled water and digested in an enzyme mixture of 1% cellulase RS (Yakult Pharmaceutical Industry Co., Ltd. Tokyo, Japan) and 1% pectolyase Y-23 (Yakult Pharmaceutical Industry Co., Ltd. Tokyo, Japan) in citric buffer (0.01 mM citric acid-sodium citrate, pH 4.5) at 28 °C for 1.0–1.5 h. The digested root tips were washed by double distilled water and transferred to a glass slide, and then mashed thoroughly with the fixative by using fine-pointed forceps. Then, the slides were dried over the flame of an alcohol lamp. The slides with abundant division cells and well-spread metaphase chromosomes were selected using an Olympus BX51 phase contrast microscope, and then stored at -20 °C until use.

### ﻿CPD staining

CPD staining was performed following the procedure indicated by She et al. (2006). In brief, the chromosome preparations were sequentially treated with RNase A and pepsin and then stained with a mixture of 0.6 µg·mL^-1^ PI and 3 µg·mL^-1^DAPI in a 30% (v/v) solution of Vectashield H100 (Vector Laboratories, Burlingame, US) for more than 30 min. Chromosome spreads were observed using an Olympus BX60 epifluorescence microscope with UV and green excitation filters. Images were captured and merged using a cooled CCD camera (CoolSNAP EZ; Photometrics, Tucson, US) controlled by METAMORPH software (Molecular Devices, California, US).

### ﻿Fluorescence *in situ* hybridization

The probe that was used to detect the 26S-5.8S-18S rRNA gene was a 9.04-kb 45S rDNA insert from tomato (see She et al. 2006), which was labeled with biotin-16-dUTP using Nick Translation Kit (Roche Diagnostics, Mannheim, Germany).

FISH with the 45S rDNA probe was conducted on the slides previously stained by CPD. The stained slides were washed in 2× SSC, twice for 15 min each, dehydrated in a graded ethanol series (70%, 90%, and 100%), air-dried at room temperature. Hybridization was performed as described by She et al. (2006). The biotin-labeled probe was detected by Fluorescein Avidin D (Vector Laboratories, Burlingame, USA). The chromosomes were counterstained and mounted with 3 µg mL^−1^DAPI in 30% (v/v) solution of Vectashield H-1000, and observed using the epifluorescence microscope mentioned above. Images were captured digitally using METAMORPH software with UV and blue excitation filters for DAPI and fluorescein, respectively.

### ﻿Karyotype analysis

Karyotype analysis followed the methodology as described by She et al. (2015). For each species, five metaphase cells whose chromosomes dispersed and condensed moderately (not reaching the maximum degree of condensation but not having decondensed terminals) were selected for measuring the length of long arm (L) and short arm (S) of each chromosome and the length of each fluorochrome band in a chromosome complement. Five metaphase cells with the maximum degree of condensation were used for measuring the absolute length of each chromosome. For the numeric characterization of the karyotypes the following parameters were calculated: (1) chromosome relative length (RL, % of haploid complement); (2) arm ratio (AR = L/S); (3) total chromosome length of the haploid complement (TCL; i.e. the karyotype length); (4) mean chromosome length (C); (5) size of the fluorochrome band (expressed as percentage of the karyotype length); (6) percent distance from the centromere to the rDNA site; (7) mean centromeric index (CI); (8) Four different karyotype asymmetry indices including coefficient of variation (CV) of centromeric index (CV_CI_), coefficient of variation (CV) of chromosome length (CV_CL_), mean centromeric asymmetry (M_CA_) and Stebbins’ asymmetry category. The meaning and calculation formulae of these asymmetry indices were given in Paszko (2006) and Peruzzi and Eroglu (2013). The arm ratio was used to classify chromosomes following the Levan’s system (Li and Chen 1985). The chromosomes were arranged in order of decreasing length except those of *C.sativus* which were organized according to the chromosome nomenclature as described by Han et al. (2008). Idiograms were drawn quantitatively based on the dataset of chromosome measurements as well as the position and size of fluorochrome bands and rDNA-FISH signals.

To visualize karyotype asymmetry relationships among the eight species, bidimensional scatter diagrams for these species with M_CA_ vs. CV_CL_ were plotted. To determine the karyological relationships among the eight species, a principal coordinate analysis (PCoA) using Gower’s similarity coefficient were performed based on six quantitative parameters (*x*, 2*n*, TCL, M_CA_, CV_CL_, CV_CI_) according to the proposal by Peruzzi and Altınordu (2014). Statistical analyses were performed with Statistica for Windows 10.0, and PCoA scatter plot was generated.

## ﻿Results

### ﻿General karyotype features

**Figure 1.**
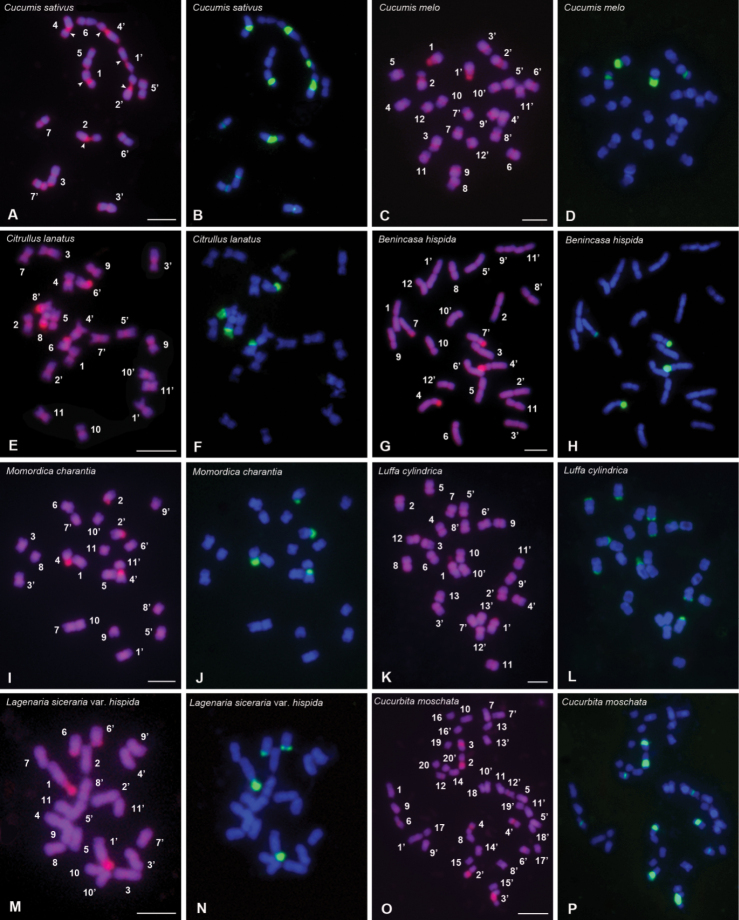
Mitotic chromosomes from *C.sativus* (**A, B**), *C.melo* (**C, D**), *C.lanatus* (**E, F**), *B.hispida* (**G, H**), *M.charantia* (**I, J**), *L.cylindrica***(K, L**), L.sicerariavar.hispida (**M, N**) and *C.moschata* (**O, P**) stained using CPD staining and sequential FISH with biotin-labelled 45S rDNA probe. **A, C, E, G, I, K, M** and **O** are the chromosomes stained using CPD. The chromosome numbers are designated by karyotyping. **B, D, F, H, J, L, N** and **P** are the chromosomes displaying 45S rDNA signals (green). The total DNA was counterstained using DAPI (blue). Scale bars: 10 µm.

**Figure 2.**
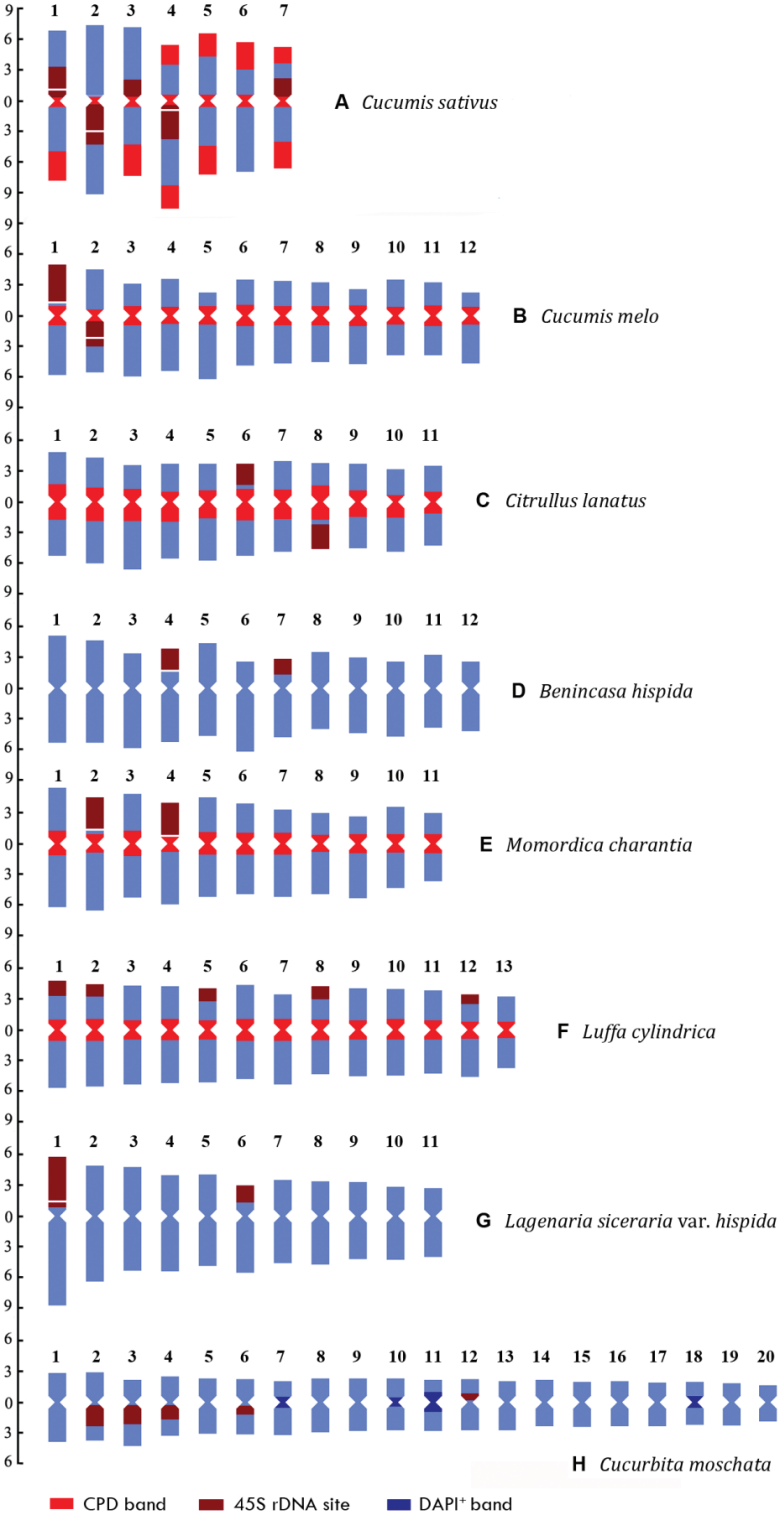
Idiograms of the eight species that display the chromosome measurements, and the position and size of the fluorochrome bands and 45S rDNA FISH signals. **A, B, C, D, E, F, G** and **H** indicate *C.sativus*, *C.melo*, *C.lanatus*, *B.hispida*, *M.charantia*, *L.cylindrica*, L.sicerariavar.hispida and *C.moschata*, respectively. The ordinate scale on the left indicates the relative length of the chromosomes (i.e. % of haploid complement). The numbers at the top indicate the serial number of chromosomes.

Using the EMF method, dispersed and morphologically well differentiated mitotic metaphase chromosomes were obtained and used for karyotyping (Fig. 1). The metaphase chromosomes with the maximum condensation degree were not very appropriate for karyotyping because of the reduction of morphological discrimination, but were suitable for the measurement of TCLs because of the comparability of TCLs between species (Suppl. material 2). The detailed karyotype features and the nuclear DNA contents of the eight species are summarized in Table 1. The measurement data of the chromosomes of each species are given in Suppl. material 3. The distribution of fluorochrome bands and 45S rDNA sites are presented in Table 2. Idiograms displaying the chromosome measurements, as well as the position and size of fluorochrome bands and 45S rDNA FISH signals are illustrated in Figure 2.

**Table 1.** Karyotype parameters of the eight Cucurbitaceae crops.

Species	KF	Genome size	TCL ± SE (μm)	C (μm)	RRL	CI±SE	CV_CI_	M_CA_	CV_CL_	St
*Cucumissativus*	2n = 14 = 12m (4SAT) + 2sm (2SAT)	367 Mb (Huang et al. 2009)	24.36 ± 1.47	3.48	11.88–16.52	44.56 ± 4.94	11.09	10.88	12.62	1A
*Cucumismelo*	2n = 24 = 16m (4SAT) + 8sm	450 Mb (Garcia-Mas et al. 2012)	33.34 ± 3.21	2.78	6.87–10.72	39.87 ± 6.37	15.99	20.27	14.51	2A
*Citrulluslanatus*	2n = 22 = 20m + 2sm	425 Mb (Guo et al. 2013)	24.94 ± 1.94	2.27	7.78–10.44	42.46 ± 3.41	8.03	15.08	10.92	1A
*Benincasahispida*	2n = 24 = 16m (2SAT) + 8sm	913 Mb (Xie et al. 2019a)	55.93 ± 4.06	4.66	6.78–10.44	41.33 ± 6.20	14.99	17.33	12.67	2A
*Momordicacharantia*	2n = 22 = 20m(4SAT) + 2sm	339 Mb (Urasaki et al. 2016)	21.31 ± 0.85	1.94	6.64–11.63	42.47 ± 4.60	10.83	15.06	17.76	2A
*Luffacylindrica*	2n = 26 = 26m	656 Mb (Wu et al. 2020)	43.75 ± 2.16	3.36	7.03–10.41	45.35 ± 2.73	6.01	8.86	9.66	1A
Lagenariasicerariavar.hispida	2n = 22 = 20m(2SAT) + 2sm	334 Mb (Achigan-Dako et al. 2008)	28.73 ± 1.69	2.61	6.67–14.52	41.94 ± 3.35	8.00	15.54	24.98	1B
*Cucurbitamoschata*	2n = 40 = 38m + 2sm	372 Mb (Sun et al. 2017)	38.15 ± 2.55	1.91	3.40–6.63	43.82 ± 3.11	7.11	12.37	18.61	2A

Notes: KF, karyotype formula; Genome size, nuclear DNA content of haploid (Values taken from previous reports and the genotypes used in DNA measurements are not necessarily identical to those in this study); TCL, total chromosome length of the haploid complement (i.e. karyotype length); C, mean chromosome length; RRL, ranges of chromosome relative length; CI, mean centromeric index; CV_CI_, CV_CL_, Coefficient of variation of the centromeric index and chromosome length, respectively; M_CA_, Mean centromeric asymmetry; St, the karyotype asymmetry category of Stebbins.

**Table 2.** The distribution of fluorochrome bands and rDNA sites in the eight Cucurbitaceae crops.

Species	Fluorochrome bands				Number (pairs) and location of 45S rDNA sites^†＃^
	Type	Distribution^†^	Amount (%)^‡^	Band size (mean)^§^	
*Cucumissativus*	CPD	All 45S sites	9.86	1.74–3.93 (2.78)	Five: 1, 3, 7S-PROX (22.06%, 12.04%, 17.41%), 2, 4L-PROX(21.53%, 15.98%)
		All CENs	13.89	0.74–1.74 (1.41)	
		1, 3, 4, 5, 7L-TERs; 4, 5, 6, 7S-TERs	21.98	1.64–3.00 (2.49)	
*Cucumismelo*	CPD	All 45S sites	6.00	2.42–3.59 (3.00)	Two: 1S-TER(27.03%), 2L-PROX(22.12%)
		All CENs, PCENs	21.62	1.18–2.04 (1.80)	
*Citrulluslanatus*	CPD	All 45S sites	4.52	2.15–2.37 (2.26)	Two: 6S-TER(41.44%), 8L-TER(48.18%)
		All CENs, PCENs	31.25	2.11–3.50 (2.84)	
*Benincasahispida*	CPD	All 45S sites	3.77	1.51–2.26 (1.89)	Two: 4, 7S-TER(41.38%, 46.43%) Two: 2S-TER(27.41%), 4S
*Momordicacharantia*	CPD	All 45S sites	6.95	3.28–3.67 (3.48)	
		All CENs, PCENs	25.72	1.89–2.98 (2.34)	
*Luffacylindrica*	CPD	All 45S sites	6.58	1.03–1.83 (1.32)	Five: 1, 2, 5, 8, 12S-TER(61.27%, 71.43%, 69.42%, 70.87%, 70.19%)
		All CENs, PCENs	25.89	1.70–2.21(1.99)	
Lagenariasicerariavar.hispida	CPD	All 45S sites	6.77	1.89–4.89 (3.39)	Two: 1S-TER(15.03%), 6S-TER(47.19%)
*Cucurbitamoschata*	CPD	All 45S sites	6.60	0.68–1.99 (1.32)	Five: 2, 3, 4, 6L-PROX(27.03%, 21.26%, 20.10%, 13.44%), 12S-PROX(15.38%)
	DAPI ^+^	7, 10, 11, 18-PCENs (post-FISH)	4.88	0.83–1.96 (1.22)	

^†^ S and L represent short and long arms, respectively; CEN, PCEN, PROX and TER represent centromeric, pericentromeric, proximal, terminal position, respectively; figures ahead of the positions are the designations of the chromosome pair involved. ^‡^ Amount of bands in the genome expressed as percentage of the karyotype length. ^§^ The percentage of the size of the bands of each chromosome pair in relation to the karyotype length. ^＃^ The percentages in square brackets are the percentage distance from centromere to the rDNA site (*di* = *d* × 100/*a*; *d* = distance of starting point of terminal sites judged by CPD bands or center of non-terminal sites judged by FISH signals from the centromere, *a* = length of the corresponding chromosome arm).

The diploid chromosome numbers are 2*n* = 2*x* = 14 for *C.sativus*, 2*n* = 2*x* = 22 for *C.lanatus*, *M.charantia* and L.sicerariavar.hispida, 2*n* = 2*x* = 24 for *C.melo* and *B.hispida*, 2*n* = 2*x* = 26 for *L.cylindrica*, and 2*n* = 2*x* = 40 for *C.moschata* (Table 1). According to the classification of Lima-de-Faria (1980), the metaphase chromosomes of *B.hispida* are of medium size with a mean chromosome length of 4.66 μm and a TCL of 55.93 μm, while those of the other seven species are of small size with a mean chromosome length between 1.91 μm (*C.moschata*) and 3.48 μm (*C.sativus*) and a TCL between 21.31 μm (*M.charantia*) to 38.15 μm (*C.moschata*). The TCLs of the eight species are basically in proportion to the nuclear DNA contents reported (Table 1). The smallest RRL (range of relative length) is observed in *C.sativus* (11.88~16.52), while the largest RRL is showed in L.sicerariavar.hispida (6.67~14.52). That is, *C.sativus* and L.sicerariavar.hispida exhibited the smallest and the largest variation in chromosome length, respectively. The mean centromeric index (CI) of the chromosome complements varied between 45.35 ± 2.73 (*L.cylindrica*) and 39.87 ± 6.37 (*C.melo*). That is, *L.cylindrica* and *C.melo* are characterized by the smallest and the largest level of variation in the centromeric index, respectively.

The karyotypes are composed of only metacentric (m) chromosomes (*L.cylindrica*) or metacentric and submetacentric (sm) chromosomes (the other seven species) (Table 1; Suppl. material 3; Fig. 2). As a whole, metacentric chromosomes are the most common form of chromosomes in the complements of the eight species studied, representing 86.60% of all chromosomes. The chromosome pairs 1, 2 and 4 in *C.sativus*, pairs 1 and 2 in *C.melo*, pair 4 in *B.hispida*, pairs 2 and 4 in *M.charantia*, and pair 1 in L.sicerariavar.hispida are satellite chromosomes (SATs) with secondary constrictions inside or in close proximity to the 45S rDNA sites (Figs 1A, C, G, I, M, 2A, B, D, E, G).

The four different karyotype asymmetry indices are given in Table 1. Among these indices, CV_CI_ is the measure of the heterogeneity of centromere position, M_CA_ characterizes the intrachromosomal asymmetry, and CV_CL_ measures the interchromosomal asymmetry (Peruzzi and Eroglu 2013; Astuti et al. 2017). The ranges of CV_CI_, M_CA_ and CV_CL_ are as follow: CV_CI_ = 6.01 (*L.cylindrica*)-15.99 (*C.melo*), M_CA_ = 8.86 (*L.cylindrica*)-20.27 (*C.melo*), CV_CL_ = 9.66 (*L.cylindrica*)-24.98 (L.sicerariavar.hispida). The M_CA_ values reveal that *L.cylindrica* and *C.melo* have the lowest and the highest intrachromosomal asymmetry, respectively. The CV_CL_ values reveal that *L.cylindrica* and L.sicerariavar.hispida have the least and the most asymmetric karyotype among the eight species in terms of interchromosomal asymmetry. According to the classification of Stebbins (1971), these karyotypes fall into 1A, 1B or 2A categories. That is, the karyotypes of all species studied are rather symmetric.

**Figure 3.**
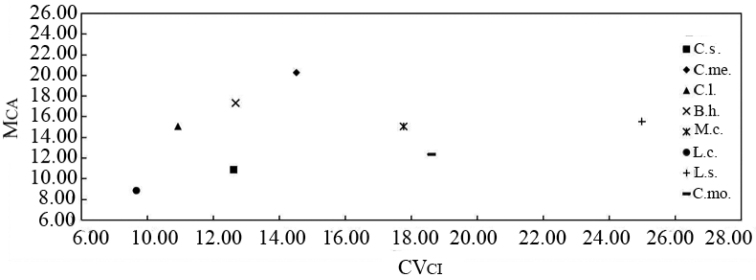
Bidimensional scatter plot of M_CA_ vs. CV_CL_ for the eight Cucurbitaceae species. C.s., C.me., C.l., B.h., M.c., L.c., L.s., and C.mo. represent *C.sativus*, *C.melo*, *C.lanatus*, *B.hispida*, *M.charantia*, *L.cylindrica*, L.sicerariavar.hispida and *C.moschata*, respectively.

The karyotype asymmetry relationships among the eight species that are expressed by means of bidimensional scatter plot of M_CA_ vs. CV_CL_ are illustrated in Figure 3. It is evident that the karyotype structure of these species can be discriminated by this couple of parameters. As depicted in the scatter plot, *L.cylindrica* is the most symmetric karyotype in terms of both intra- and inter-chromosomal asymmetry, while *C.melo* and L.sicerariavar.hispida are the most asymmetric karyotypes in terms of intra- and inter-chromosomal index, respectively (Fig. 3).

**Figure 4.**
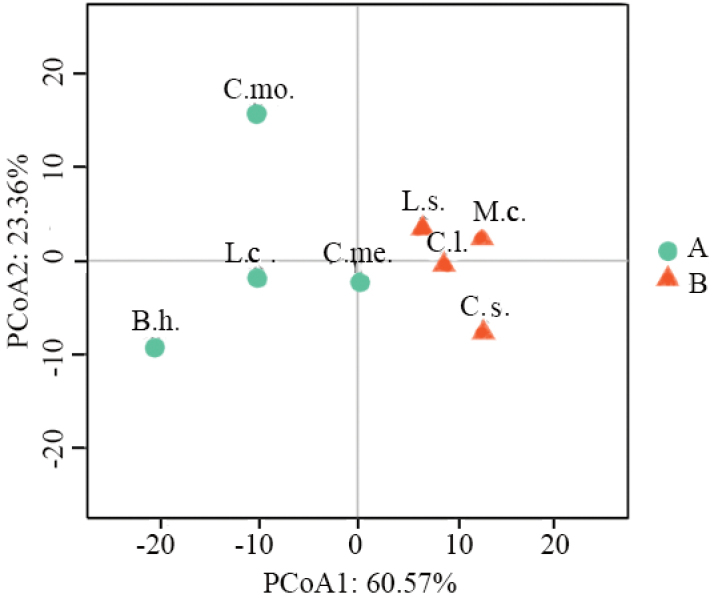
PCoA for the eight Cucurbitaceae species based on *x*, 2*n*, TCL, M_CA_, CV_CL_ and CV_CI_. C.s., C.me., C.l., B.h., M.c., L.c., L.s., and C.mo. represent *C.sativus*, *C.melo*, *C.lanatus*, *B.hispida*, *M.charantia*, *L.cylindrica*, L.sicerariavar.hispida and *C.moschata*, respectively. PCoA1 reflects the original data characteristics before the dimensionality reduction of 60.57%. PCoA2 reflected the character of the original data before the dimensionality reduction of 23.36%. The sum of the two percentages is 83.93%, indicating that the two-dimensional coordinate system can reflect the characteristics of 83.93% of the original data.

Karyological relationships among the studied species revealed by PCoA based on six karyological parameters are illustrated in Figure 4. The PCoA scatter plot shows that the eight species can be divided into two groups along the direction of PCoA1: L.sicerariavar.hispida, *C.lanatus*, *M.charantia* and *C.sativus* in one group with the former three species closely clustering together, *C.melo*, *L.cylindrica*, *B.hispida* and *C.moschata* in another group in which *C.melo* occupies the middle position of the two groups and *C.moschata* occupies the most isolated position (Fig. 4).

### ﻿Fluorochrome banding patterns and 45S rDNA sites

CPD staining and DAPI counterstaining revealed distinct heterochromatin differentiation among the eight species (Figs 1, 2; Suppl. material 2; Table 2). In each species, all the chromosomal regions corresponding to the 45S rDNA sites which were confirmed by the subsequent FISH with the 45S rDNA probe showed CPD bands (Fig. 1A, C, E, G, I, K, M, O). All (peri) centromeric regions in *C.sativus*, *C.melo*, *C.lanatus*, *M.charantia* and *L.cylindrica* displayed CPD bands (Fig. 1A, C, E, I, K; Suppl. material 2: fig. S1A–C, E, F), while those in *B.hispida*, L.sicerariavar.hispida and *C.moschata* did not display CPD bands (Fig. 1G, M, O; Suppl. material 2: fig. S1D, G, H). Particularly, in *C.sativus*, the terminals of the short arms of pairs 4, 5, 6 and 7 and the long arms of pairs 1, 3, 4, 5 and 7 displayed CPD bands (Figs 1A, 2A). In *C.moschata*, after the FISH procedure, DAPI counterstaining showed pericentromeric DAPI^+^ bands (called post-FISHDAPI^+^ bands) on chromosome pairs 7, 10, 11 and 18 (Fig. 1P). The total amount of the (peri)centromeric CPD bands in *C.sativus*, *C.melo*, *C.lanatus*, *M.charantia* and *L.cylindrica* are 13.89%, 21.62%, 31.25%, 25.72% and 25.89% of the karyotype length, respectively (Table 2; Suppl. material 3). The total amount of the terminal CPD bands in *C.sativus* is 21.98% of the karyotype length (Table 2; Suppl. material 3). The total amount of post-FISHDAPI^+^ bands in relation to the karyotype length is 4.88% in *C.moschata* (Table 2; Suppl. material 3). The size of the rDNA CPD bands, non-rDNA CPD bands and post-FISHDAPI^+^ bands varied among the chromosome pairs (Fig. 2; Table 2; Suppl. material 3).

FISH with the 45S rDNA probe onto the chromosomes previously stained by CPD is presented in Figure 1. The number and location of 45S rDNA sites are summarized in Table 2, and illustrated in Figure 2. There are obvious differences in number, size and location among the eight species (Table 2). Two 45S loci were detected in *C.melo*, *C.lanatus*, *B.hispida*, *M.charantia* and L.sicerariavar.hispida, and five 45S loci were detected in *C.sativus*, *L.cylindrica* and *C.moschata* (Figs 1B, D, F, H, J, L, N, P, 2). There were twenty-five 45S rDNA loci in the eight taxa, of which 14 (accounting for 56%) were located at the terminals and 11 (accounting for 44%) were located in the proximal regions of the respective chromosome arms (Fig. 2; Table 2). In *C.sativus*, the five 45S rDNA loci are located in the proximal regions of the short arms of chromosome pairs 1, 3 and 7 and the long arms of pairs 2 and 4. The 45S rDNA sites of pairs 1, 2 and 4 are major loci in which secondary constrictions appear in prophase and prometaphase cells, while those of pairs 3 and 7 are minor loci (Figs 1A, B, 2A). In *C.melo*, one 45S locus is distally located on the short arms of pair 1 and occupies the majority of the arms, another 45S locus is located in the proximal regions of the long arms of pair 2 (Figs 1C, D, 2B). There are secondary constrictions on the proximal side of the 45S locus of pair 1 and inside the 45S locus of pair 2. In *C.lanatus*, the two 45S rDNA loci are terminally located in the short arms of pair 6 and the long arms of pair 8, respectively (Figs 1E, F, 2C). In *B.hispida*, one 45S locus is located at the terminals of the short arms of pair 4 beside which secondary constrictions occur, another 45S locus is terminally located in the short arms of pair 7 (Figs 1G, H, 2D). The 45S loci of both *C.lanatus* and *B.hispida* account for more than half of the respective arms (Fig. 2C, D; Table 2). In *M.charantia*, one 45S locus is terminally located on the short arms of pair 2 and occupies the majority of the arms, another locus occupies the entire short arms of pair 4 (Figs 1I, J, 2E; Table 2). There are secondary constrictions beside the two 45S loci (Figs 1I, 2E). The five 45S loci of *L.cylindrica* are relatively small and located at the terminals of the short arms of pairs 1, 2, 5, 8 and 12 (Figs 1K, L, 2F). In L.sicerariavar.hispida, one 45S locus is located at the terminals of the short arms of pair 1 which accounts for the majority of the arms and produces secondary constrictions in the interior of the sites, another locus is situated at the terminals of the short arms of pair 6 (Figs 1M, N, 2G). In *C.moschata*, three major 45S loci are located in the proximal regions of the long arms of pairs 2, 3 and 4, respectively, two minor loci are proximally placed on the long arms of pair 6 and the short arms of pair 12, respectively (Figs 1O, P, 2H). In particular, the size of the hybridization signals and CPD bands of the 45S rDNA sites of pair 3 vary significantly between two homologous chromosomes (Fig. 1O, P), indicating the heterozygosity of the *C.moschata* accession analyzed in this study.

We find that all mitotic chromosomes of *C.sativus* can be precisely identified by the combination of the 45S rDNA FISH signals and terminal CPD bands (Figs 1A, B, 2A). The features of each chromosome pair of *C.sativus* are as follows. Chromosome 1 has strong 45S rDNA signal in the proximal region of the short arm and terminal CPD band on the long arms. Chromosome 2 has strong 45S rDNA signal in the proximal region of the long arm and is devoid of CPD band at the terminals of both arms. Chromosome 3 has weak 45S rDNA signal in the proximal region of the short arm and terminal CPD band on the long arm. Chromosome 4 has strong 45S rDNA signal in the proximal region of the long arm and terminal CPD bands on both arms. Chromosome 5 has terminal CPD bands on both arms and is devoid of 45S rDNA signal. Chromosome 6 has terminal CPD band on the short arm and is devoid of 45S rDNA signal. Chromosome 7 has weak 45S rDNA signal in the proximal region of the short arm and terminal CPD bands on both arms.

## ﻿Discussion

### ﻿Karyotype features and 45S rDNA patterns

Precise chromosome measurement is essential for accurate karyotype analysis. Chromosomes should have morphologically distinct primary constrictions and clearly defined boundaries; otherwise, it is difficult to determine the length of chromosome arms and, consequently, to calculate chromosomal parameters (Kadluczka and Grzebelus 2021). In our previous cytogenetic investigations, it was found that the morphological differentiation of mitotic chromosomes depended on the degree of condensation (She et al. 2006, 2015, 2017, 2020; She and Jiang 2015). When condensation is insufficient, the terminals of chromosome arms may be still in the decondensation state, and then the boundary of chromosomes is not clear. However, when chromosomes condensed to the maximum, the morphological differences between chromosomes decreased. Therefore, it is important to select metaphase chromosomes with moderate degree of condensation for identification and measurement of chromosomes. This is especially true for species with small chromosomes. In addition, the landmarks produced by CPD staining and rDNA FISH facilitated chromosome identification (She et al. 2006, 2015, 2017, 2020; She and Jiang 2015). In this study, well morphologically differentiated metaphase chromosomes of the eight Cucurbitaceae crops were prepared using the EMF method and characterized by fluorochrome banding and 45S rDNA-FISH. Detailed karyotypes of the eight species were established with a combined dataset consisted of chromosome measurements, fluorochrome bands and 45S rDNA FISH signals. Furthermore, four different karyotype asymmetry indices of the eight species were simultaneously measured for the first time. Therefore, the newly constructed karyotypes of these species are more accurate, informative and comparable.

The current karyotype of *C.sativus* differs in chromosome size and the classification of chromosome morphotype from some previously reported karyotypes of this species. The range of chromosome size and TCL detected in our study are similar to those of Trivedi and Roy (1970), but much larger than those of Chen et al. (1998) and Koo et al. (2002). The karyotype formula obtained here is similar to those reported by Li (1989), Chen et al. (1998), Koo et al. (2002) and Waminal and Kim (2012) which also consisted of 12 m and 2 sm, but different from those reported by Trivedi and Roy (1970), Han et al. (2008) and Zhang et al. (2015b) in which all chromosomes were metacentric, and that reported by Hoshi et al. (1998) which was comprised of 10 m and 4 sm. In the current karyotype, chromosome 2 is the longest and chromosome 4 is submetacentric, while in the karyotype reported by Han et al. (2008), chromosome 3 is the longest and chromosome 4 is metacentric. We identified three pairs of satellite chromosomes (pairs 1, 2 and 4) in *C.sativus* by means of CPD staining. Secondary constructions had been observed in *C.sativus* by several investigators (Bhaduri and Bose 1947; Trivedi and Roy 1970; Ramachandran and Seshadri 1986; Li 1989), but the number of secondary constrictions have not been reliably confirmed using the conventional staining. The 45S rDNA pattern of *C.sativus* including the number, position and size of 45S sites revealed by us is consistent with those reported by Han et al (2008).

The chromosome size of *C.melo* detected by us is similar to that of Li (1989), but differs considerably from that of Trivedi and Roy (1970). Our karyotype of *C.melo* which is composed of both m and sm chromosomes is similar to that of Zhang et al. (2015a), but differs from those of Liu et al. (2010) and Li (1989) in which one or more pairs of subterminal (st) chromosomes were involved. In current study, all centromeres of *C.melo* were marked by the CPD bands, enabling the arm ratio of each chromosome to be accurately measured. The number and position of the 45S sites of *C.melo* detected in this study is coincident with the previous reports (Zhang et al. 2015a; Zhang et al. 2016). In our karyotype of *C.melo*, the chromosome pairs bearing the 45S sites are designated as pairs 1 and 2 according to the descending order of the length. They should correspond to chromosome pairs 4 and 10 in the karyotype of Zhang et al. (2015a), respectively. In addition, the arms of pair 2 that have 45S sites are identified as the long arms in our study, instead of the short arms as described by Zhang et al. (2015a).

The TCL of *C.lanatus* detected by us is larger than those of Waminal et al. (2011) and Trivedi and Roy (1970). Our karyotype formula of *C.lanatus* is accordance with those described by Li (1989) and Li et al. (2007), and slightly differs from that reported by Waminal et al. (2011) in which 14 m and 8 sm were involved. The 45S rDNA sites situated on chromosome pairs 6 and 8 of *C.lanatus* in our study should correspond to those mapped on pairs 8 and 4 by Ren et al. (2012), Guo et al. (2013) and Li et al. (2016), respectively. However, the rDNA-bearing arms of pair 8 was designated as the long arms in our study rather than the shorts arms (Ren et al. 2012; Guo et al. 2013; Li et al. 2016).

The karyotype formula of *B.hispida* obtained in our study resemble those reported by Li (1989) and Xu et al. (2007), but varies significantly from that of Waminal et al. (2011). The TCL of *B.hispida* detected by us is similar to that of Li (1989), but larger than that of Waminal et al. (2011). Our study demonstrates that both of the two 45S rDNA sites in *B.hispida* are located at the terminals of the short arms of the respective chromosomes instead of subtelomeric or interstitial regions of the respective short arms (Xu et al. 2007; Waminal et al. 2011), which is consistent with the result of Xie et al. (2019b).

Our karyotype formula of *M.charantia* is similar to that reported by Li (1989) and slightly different from those of Waminal and Kim (2012) and Li et al. (2007). The TCL detected by us is larger than that of Waminal and Kim (2012), and smaller than that of Li (1989). In this study, sequential CPD staining and 45S rDNA FISH reveal that the two 45S rDNA loci occupy the majority or the entire length of the respective short arms, providing a more accurate mapping of the 45S rDNA sites in *M.charantia* than previous studies (Li et al. 2007; Waminal and Kim 2012; Xie et al. 2019b).

The karyotype formula of *L.cylindrica* obtained by us is in accordance with that of Xu et al. (2007), but slightly differing from those of Li (1989) and Waminal and Kim (2012). The TCL of *L.cylindrica* detected in our study is similar to that of Li (1989), but is much larger than that of Waminal and Kim (2012). Our study demonstrates that all the five 45S rDNA loci are terminally located on the short arms of five chromosome pairs, being consistent with the result of Waminal and Kim (2012), but different from the result of Xu et al. (2007) in which the positions of the five 45S loci were identified as subtelomeric regions.

Our karyotype formula of L.sicerariavar.hispida is coincident with that of Li (1989), but differs from the karyotype of *L.siceraria* reported by Waminal and Kim (2012). The TCL of L.sicerariavar.hispida detected in this study is slightly smaller than that of Li (1989), but much larger than the TCL of *L.siceraria* detected by Waminal and Kim (2012). Our study reveals that the number of 45S rDNA sites in L.sicerariavar.hispida is the same as in *L.siceraria*, and the rDNA sites are also located at the terminals of the short arms of two chromosome pairs (Waminal and Kim 2012; Li et al. 2016; Xie et al. 2019b).

The karyotype formula of *C.moschata* constructed by us consists of only m and sm chromosomes, being similar to that reported by Waminal et al. (2011). However, it is considerably different from those of Li (1989) and Xu et al. (2007) in which, except for m and sm chromosomes, four and eight st chromosomes were involved, respectively. The chromosome size of *C.moschata* detected by us is similar to that of Li (1989), but much larger than that of Waminal et al. (2011). In our study, the locations and sizes of the five 45S rDNA loci of *C.moschata* are determined accurately by the rDNA CPD bands, demonstrating that all the five 45S loci are at proximal localization instead of terminal localization (Xu et al. 2007; Waminal et al. 2011).

The discrepancies in karyotype feature and 45S rDNA pattern between our results and the previous reports are probably due to differences in the accessions analyzed, the condensation level of measured chromosomes, and the difficulty in identifying chromosomes using the mitotic chromosome spreads of lower quality in the previous studies.

### ﻿Genome differentiation between species

The total chromosome length of the haploid complement (TCL) can be used as a proxy for genome size (Levin 2002). Previous studies found that the correlations between TCL and DNA content typically exceeded *r* = 0.85 within species, between congeneric species and among species in related genera (Levin 2002; Carta and Peruzzi 2016). In our study, the TCL of each of the eight taxa was measured using the chromosomes with the highest degree of condensation (She et al. 2015), and therefore, the TCLs of these species can be well comparable with each other. The correlation analysis using the SPSS 25.0 software (Suppl. material 4) reveals a high correlation between the difference in TCL and the change in nuclear DNA content within the eight taxa (*r* = 0.899, *p* < 0.01), providing new evidence of the feasibility of comparing genome size based on TCL values among species of related genera. For example, whether according to TCL or nuclear DNA content, *B.hispida* was the largest genome and *M.charantia* was the smallest genome, and *L.cylindrica* genome was about twofold larger than *M.charantia* genome (Urasaki et al. 2016; Xie et al. 2019a; Wu et al. 2020). However, it was also found that TCL and DNA content values were incompletely proportional to each in some other cases. For example, the DNA contents of *C.sativus* and *C.moschata* are almost equal (Huang et al. 2009; Sun et al. 2017), but the TCL of *C.moschata* is 1.6 times as much as that of *C.sativus*. Several studies have revealed that total chromosome volume, instead of TCL could be a descriptor of chromosome size, and more suitable to reflect genome size (Kramer et al. 2021; Mehravi et al. 2022). This may account for the inconsistency. Increase in genome size may, in general, be attributed to transposable element amplification and to polyploidization. Genome sequencing studies revealed that repeat expansion led to large genome size in cucurbits (Garcia-Mas et al. 2012; Xie et al. 2019a; Wu et al. 2020). For example, *B.hispida* genome did not have any recent lineage-specific whole-genome duplication as other sequenced species in the tribe Benincaseae including *C.sativus*, *C.melo*, *C.lanatus* and *L.cylindrica*, the substantial accumulation of transposable elements and especially LTR retrotransposons contributes greatly to the large genome size of this species (Xie et al. 2019a; Wu et al. 2020).

The differences in CPD and DAPI^+^ bands, with regard to presence, position and size, reveal distinct heterochromatin differentiation among the eight cucurbits studied. CPD staining reveals the occurrence of (peri)centromeric GC-rich heterochromatin in *C.sativus*, *C.melo*, *C.lanatus*, *M.charantia* and *L.cylindrica*, and terminal GC-rich heterochromatin in *C.sativus*. (Peri)centromeric and terminal GC-rich heterochromatin was previously detected in *C.sativus* using CMA/DAPI staining (Plader et al. 1998). According to the recent classification of Cucurbitaceae, *Cucumis*, *Benincasa*, *Citrullus* and *Lagenaria* Seringe, 1825 belong to tribe Benincaseae, and *Luffa* Miller, 1754, *Momordica* Linnaeus, 1753 and *Cucurbita* Linnaeus, 1753 belong to tribe Luffeae, Joliffieae and Cucurbiteae, respectively (Jeffrey 2005; Kocyan et al. 2007). *Cocciniagrandis* (Linnaeus) Voigt, 1845 (2*n* = 24), a species of tribe Benincaseae, showed centromeric GC-rich heterochromatin in the majority of chromosome pairs by CMA/DAPI staining (Bhowmick et al. 2012). These facts suggest that the presence of (peri)centromeric GC-rich heterochromatin is an ancestral genome feature that occurred before the divergence of Subfamily Cucurbitoideae. However, the inexistence of (peri)centromeric GC-rich heterochromatin in *B.hispida*, L.sicerariavar.hispida and *C.moschata* seems to be contradiction with this speculation. A reasonable explanation is that the (peri)centromeric GC-rich heterochromatin of these three species has undergone a reduction of GC content after speciation, resulting in the disappearance of red CPD bands (She et al. 2006, 2015). The (peri)centromeric CPD bands in *C.melo* may result from staining of the centromere-specific repeats (CmCent) whose GC contents is rather high (56–57%) (Koo et al. 2010). The terminal GC-rich heterochromatin of *C.sativus* should be differentiated during speciation because *C.melo* has not such heterochromatin. In the *C.sativus* genome, two types of tandem repeats, Type I/II and Type IV, are located in the subtelomeric regions of the majority of chromosome arms in the complement, while tandem repeat type III and centromere-specific satellite 1 (CsCent1) are located in the centromeres of each chromosome (Han et al. 2008; Ren et al. 2009; Koo et al. 2010; Zhao et al. 2011). Given that Type I/II, Type III, Type IV, and CsCent1 have higher GC content (Han et al. 2008; Koo et al. 2010), the centromeric CPD bands may result from the staining of type III and CsCent1, and the terminal CPD bands may result from the staining of Type I/II and Type IV. The FISH signals of Type I/II and Type IV were detected all but one of chromosome arms (Han et al. 2008; Ren et al. 2009), while the terminal CPD bands were only detected on 9 out of 14 chromosome arms. This discrepancy is probably attributed to the lower sensitivity of CPD staining compared with FISH technology or the difference in the accessions analyzed. The occurrence of post-FISHDAPI^+^ bands in *C.moschata* was a prominent indication of heterochromatic differentiation. DAPI^+^ bands revealed only after FISH procedure have been reported in many plant species (e.g. Morales et al. 2012; She et al. 2015), and should represent another kind of heterochromatin that is different from GC- and AT-rich heterochromatin (Barros e Silva and Guerra 2010).

The number, location and distribution of the 5S and 45S rDNA clusters in chromosomes are useful for deducing species history and phylogenetic relationship (Weiss-Schneeweiss et al. 2008). We statistically analyzed the number and position of the 45S rDNA sites from 58 species (subspecies or varieties) in Cucurbitaceae (a total sample of 64 karyotypes) that have been investigated by FISH up to now (Suppl. material 5). In the 64 karyotypes, 43 of which are of the species belonging to tribe Benincaseae (Kocyan et al. 2007), there were a total of 182 45S loci, of which 137 (accounting for 75.3%) were located in the terminal portions of chromosomes (including the sites occupying the whole arm), 41 loci in the proximal regions, and 4 in the pericentromeric or interstitial regions (Suppl. material 5); and the number of 45S rDNA sites per complement ranged from one pair up to seven pairs with the most frequent numbers of sites per karyotype being two pairs (accounting for 43.7%). Such distribution feature of 45S rDNA sites in Cucurbitaceae is basically consistent with the general distribution pattern in the entire angiosperms which shows that 45S rDNA sites occur preferentially on the short arms and in the terminal regions of chromosomes (Roa and Guerra 2012). Four of the eight species studied here, including *C.lanatus*, *B.hispida*, L.sicerariavar.hispida and *M.charantia* had similar distribution of 45S rDNA sites (owning two 45S loci of terminal position), suggesting a close relationship between these four species. This was in accord with the results based on molecular phylogenetic analyses, which revealed that there were close relationships among the genera *Citrullus*, *Benincasa* and *Lagenaria* (Zhang et al. 2018; Xie et al. 2019a; Wu et al. 2020), and that *M.charantia* was more related to *C.lanatus* than to *C.sativus* and *C.melo* (Urasaki et al. 2016). In addition, among the 43 karyotypes of tribe Benincaseae, 22 karyotypes (accounting for 51.2%) had two 45S rDNA loci of terminal position (Suppl. material 5). Based on these facts, we speculate that the ancestral progenitor of tribe Benincaseae might bear two 45S loci that were located in the terminal portions of two chromosome pairs. Very recently, the ancestral karyotype of *Cucumis* was reconstructed using comparative oligo-painting, which owns two 45S rDNA loci that located in the terminal regions of two short arms (Zhao et al. 2021). The ancestral karyotype of lineage I of *Cucumis* (2*n* = 2*x* = 24), which has two 45S rDNA loci of terminal position, evolved to *C.melo* mainly by inversions, and evolved to *C.sativus* mainly by chromosome fusions and inversions (Zhao et al. 2021).

Concerning karyotype asymmetry is one of the most popular, cheap and widely used cytotaxonomic approach. Up to now, a variety of parameters and indices for evaluating karyotype asymmetry have been proposed, including the quali-quantitative one, Stebbins category (Stebbins 1971), as well as several quantitative indices (for details and references see Paszko 2006; Peruzzi and Eroglu 2013). Critical reviews have confirmed that CV_CL_ is a powerful statistical parameter for estimating the interchromosomal asymmetry, M_CA_ is the most appropriate parameter for a measure of intrachromosomal asymmetry, and other quantitative indices are outdated, redundant, or statistically incorrect (Paszko 2006; Peruzzi and Eroglu 2013; Astuti et al. 2017). The best way in representing karyotype asymmetry relationships among taxa is by means of bidimensional scatter plot, where the two asymmetry estimators are put in the x and y axes and points represent each sample (Peruzzi et al. 2009; Peruzzi and Eroglu 2013; Dehery et al. 2020). Our results show that the karyotype asymmetry relationships among the eight Cucurbitaceae species studied can be best explained by means of the scatter plot of M_CA_ vs. CV_CL_, confirming that this couple of indices are reliable to assess chromosome asymmetry.

In order to compare karyotypes and reconstructing karyological relationships among the eight species, we applied the methodology proposed by Peruzzi and Altınordu (2014), considering six quantitative parameters (*x*, 2*n*, TCL, M_CA_, CV_CL_, CV_CI_) and subjecting them to PCoA (Dehery et al. 2020; Kadluczka and Grzebelus 2021). Our results demonstrated PCoA with the six parameters was indeed a good way to establish the karyological relationships among the eight Cucurbitaceae species because the karyological relationships among the eight taxa established by PCoA was found to be basically accordant with the phylogenetic relationships revealed by DNA sequences. In the molecular phylogenetic trees, *C.lanatus* and *L.siceraria* were very closely related, and both of them were closely to *C.melo* and *C.sativus* (Kocyan et al. 2007; Zhang et al. 2018). Genome sequencing analysis revealed that *M.charantia* was more related to *C.lanatus* than to *C.sativus* and *C.melo* (Urasaki et al. 2016). However, we also observed something different from the molecular evolutionary trees. *B.hispida*, a species with close relationship with *C.lanatus* and *L.siceraria* in the molecular phylogenetic trees (Kocyan et al. 2007; Xie et al. 2019a), was distantly separated from the two species in the PCoA scatter plot. On the whole, to infer the direction of changes of karyotype evolution in Cucurbitaceae species, karyotype asymmetry study using the multivariate quantitative approach is recommended as one of the complementary characters besides the molecular taxonomic character.

The reported basic chromosome numbers of the Cucurbitaceae family ranged from *x* = 7 to 20, with *x* = 11 a prevalent number (Carta et al. 2020). Recent comparative analyses of six cucurbit genomes reveal that the *B.hispida* genome represents the most ancestral karyotype, with the predicted ancestral genome having 15 proto-chromosomes (Xie et al. 2019a). After *B.hispida*, the 15 ancestral chromosomes were either retained or form new chromosomes through chromosome arrangements such as fusions, fissions, inversions during the speciation and evolution of later species (Xie et al. 2019a). Recent studies revealed that *C.melo* and *C.sativus* were evolved from an ancestral karyotype (*x* = 12) by large-scale inversions, centromere repositioning and chromothripsis-like rearrangement (Zhao et al. 2021), and *C.moschata* resulted from an ancient allotetraploidization event (Sun et al. 2017). Alterations in chromosome symmetry may arise through chromosome arrangements including translocations, pericentric inversions, fusions or fissions (Schubert 2007), or through removes or addition of the same amount of DNA from/to both arms of chromosomes (Levin 2002; Peruzzi et al. 2009). Although many alterations in number and structure of chromosomes as well as genome size occurred during speciation and evolution, the karyotype asymmetry of the eight species involved in this study had not changed significantly, and their karyotypes were all symmetrical. The reasons for this are worth further study.

## ﻿Conclusions

Detailed karyotypes of eight Cucurbitaceae crops, *C.sativus*, *C.melo*, *C.lanatus*, *B.hispida*, *M.charantia*, *L.cylindrica*, L.sicerariavar.hispida and *C.moschata*, were reconstructed using the dataset of chromosome measurements, fluorochrome bands and 45S rDNA FISH signals. Comparative karyotyping revealed distinct variations in the karyotypic parameters, and the patterns of fluorochrome bands and 45S rDNA sites among species. The karyological relationships among the eight taxa based on six karyological parameters was basically accordant with the phylogenetic relationships revealed by DNA sequences, indicating that karyotype asymmetry study using the multivariate quantitative approach is one of the complementary characters for inferring the direction of changes of karyotype evolution in Cucurbitaceae species.

## ﻿Competing interests

The authors have declared that no competing interests exist.

## Supplementary materials

Supplementary material 1 The plant materials Data type table (docx. file) https://binary.pensoft.net/file/807550This dataset is made available under the Open Database License
(http://opendatacommons.org/licenses/odbl/1.0/). The Open Database License (ODbL) is a
license agreement intended to allow users to freely share, modify, and use this
Dataset while maintaining this same freedom for others, provided that the original
source and author(s) are credited.Chao-Wen She, Xiang-Hui Jiang, Chun-Ping He

Supplementary material 2 CPD-stained mitotic metaphase chromosomes with the maximum condensation degree Data type figure (docx. file) Explanation note CPD-stained mitotic metaphase chromosomes with the maximum condensation degree from *C.sativus* (**A**), *C.melo* (**B**), *C.lanatus* (**C**), *B.hispida* (**D**), *M.charantia* (**E**), *L.cylindrica* (**F**), L.sicerariavar.hispida (**G**) and *C.moschata* (**H**). Scale bars: 10 µm. https://binary.pensoft.net/file/807551This dataset is made available under the Open Database License
(http://opendatacommons.org/licenses/odbl/1.0/). The Open Database License (ODbL) is a
license agreement intended to allow users to freely share, modify, and use this
Dataset while maintaining this same freedom for others, provided that the original
source and author(s) are credited.Chao-Wen She, Xiang-Hui Jiang, Chun-Ping He

Supplementary material 3 Chromosome measurements of the eight Cucurbitaceae crops obtained from five metaphases per species Data type table (docx. file) https://binary.pensoft.net/file/807552This dataset is made available under the Open Database License
(http://opendatacommons.org/licenses/odbl/1.0/). The Open Database License (ODbL) is a
license agreement intended to allow users to freely share, modify, and use this
Dataset while maintaining this same freedom for others, provided that the original
source and author(s) are credited.Chao-Wen She, Xiang-Hui Jiang, Chun-Ping He

Supplementary material 4 The correlational analysis between the difference in TCL and the change in nuclear DNA content within the eight Cucurbitaceae crops using the SPSS 25.0 software Data type table (docx. file) https://binary.pensoft.net/file/807553This dataset is made available under the Open Database License
(http://opendatacommons.org/licenses/odbl/1.0/). The Open Database License (ODbL) is a
license agreement intended to allow users to freely share, modify, and use this
Dataset while maintaining this same freedom for others, provided that the original
source and author(s) are credited.Chao-Wen She, Xiang-Hui Jiang, Chun-Ping He

Supplementary material 5 The number and position of 45S rDNA locus in Cucurbitaceae species Data type table (docx. file) https://binary.pensoft.net/file/807554This dataset is made available under the Open Database License
(http://opendatacommons.org/licenses/odbl/1.0/). The Open Database License (ODbL) is a
license agreement intended to allow users to freely share, modify, and use this
Dataset while maintaining this same freedom for others, provided that the original
source and author(s) are credited.Chao-Wen She, Xiang-Hui Jiang, Chun-Ping He
