# Supplementary material for: ﻿Comparative karyotype analysis of eight Cucurbitaceae crops using fluorochrome banding and 45S rDNA-FISH
Source: Comp Cytogenet. 2023 Feb 9;17:31–58. doi: 10.3897/compcytogen.17.99236 (PMC10252140; doi:10.3897/compcytogen.17.99236)
Supplement: Supplementary material 1 — The plant materials [file comparative_cytogenetics-17--031_article-99236__-s001.docx]

**Table S1. The plant materials**

| Species | Accessions | Source |
| --- | --- | --- |
| *Cucumis sativus* | Tangshan Qiugua | Zhengzhou Hongfeng Seed Co. Ltd, Henan Province, China |
| *Cucumis melo* | Huanong Tianbao Xianggua | Beijing San Kaihua Agricultural Technology Co., Ltd, Beijing, China |
| *Citrullus lanatus* | Huakang Xigua No.8 | Qingxian Shenhua Seed Co. Ltd, Hebei Province, China |
| *Benincasa hispida* | Hanyu Fenpi Donggua | Qingxian Chunfeng Vegetable Seed Breeding Base, Hebei Province, China |
| *Momordica charantia* | Lanshan Dabai Kugua | Lanshan Bitter Gourd Base, Hunan Province,China |
| *Luffa cylindrica* | Changsha Rou Sigua | Beijing High-tech Development Co., Ltd, Beijing, China |
| *Lagenaria siceraria* var. *hispida* | Changfeng Jingpin Huzi | Qingxian Chunfeng Vegetable Seed Breeding Base, Hebei Province, China |
| *Cucurbita moschata* | Da Mopan Nangua | Lengshuijiang Seed Co. Ltd, Hunan Province, China |
