# Supplementary material for: ﻿Comparative karyotype analysis of eight Cucurbitaceae crops using fluorochrome banding and 45S rDNA-FISH
Source: Comp Cytogenet. 2023 Feb 9;17:31–58. doi: 10.3897/compcytogen.17.99236 (PMC10252140; doi:10.3897/compcytogen.17.99236)
Supplement: Supplementary material 2 — CPD-stained mitotic metaphase chromosomes with the maximum condensation degree [file comparative_cytogenetics-17--031_article-99236__-s002.docx]

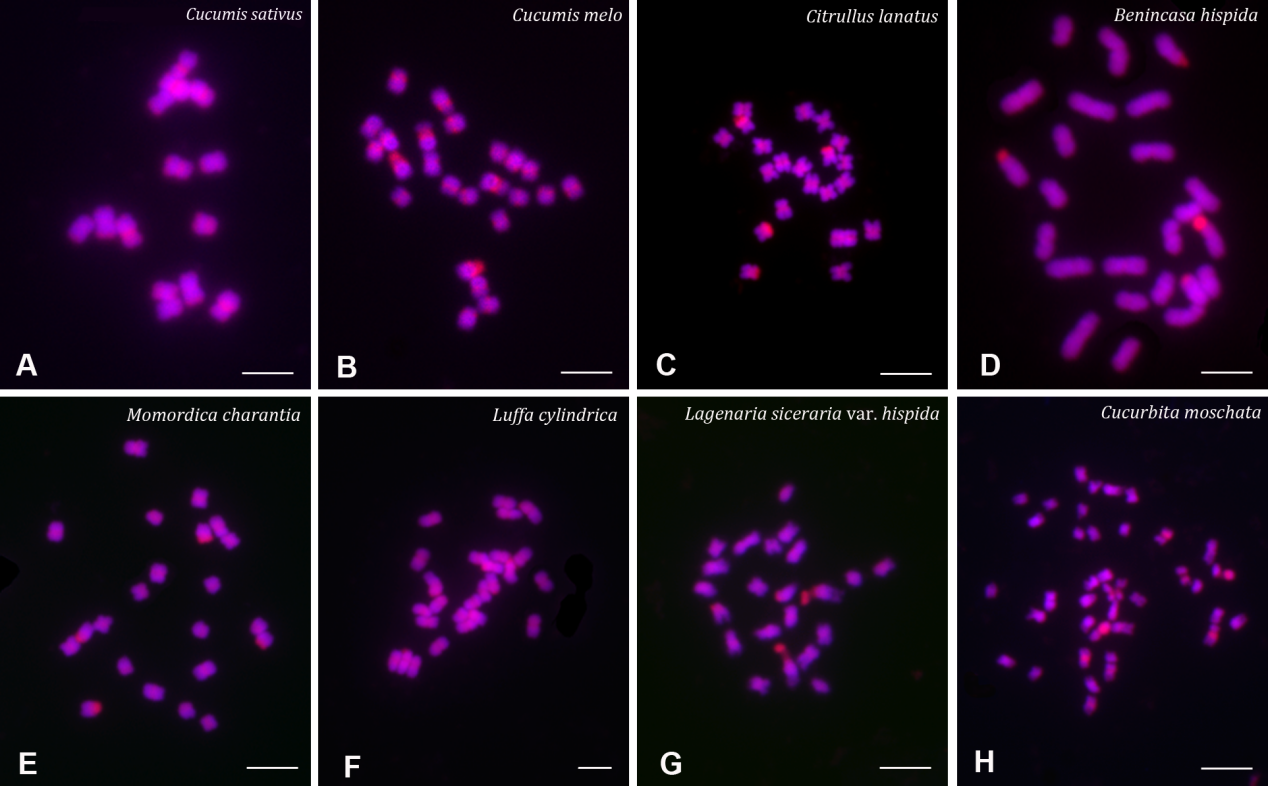


**Figure S1.** CPD-stained mitotic metaphase chromosomes with the maximum condensation degree from *C. sativus* (**A**), *C. melo* (**B**), *C. lanatus* (**C**), *B. hispida* (**D**), *M. charantia* (**E),** *L. cylindrica* **(F**), *L. siceraria* var. *hispida* **(G**) and *C. moschata* **(H**). Scale bars = 10 µm.
