# Supplementary material for: ﻿Comparative karyotype analysis of eight Cucurbitaceae crops using fluorochrome banding and 45S rDNA-FISH
Source: Comp Cytogenet. 2023 Feb 9;17:31–58. doi: 10.3897/compcytogen.17.99236 (PMC10252140; doi:10.3897/compcytogen.17.99236)
Supplement: Supplementary material 3 — Chromosome measurements of the eight Cucurbitaceae crops obtained from five metaphases per species [file comparative_cytogenetics-17--031_article-99236__-s003.docx]

**Table S2. Chromosome measurements of the eight Cucurbitaceae crops obtained from five metaphases per species.**

| Species | Chr. No. | Relative length (%) | | | Arm ratio ± SD | Type | (Peri)centromeric CPD band size^‡^ ± SD | rDNA CPD band size^‡^ ± SD | Terminal CPD band size^‡^ ±SD | |
| --- | --- | --- | --- | --- | --- | --- | --- | --- | --- | --- |
|  |  | Short arm ± SD | Long arm ± SD | Total ± SD |  |  |  |  |  |  |
|  |  |  |  |  |  |  |  |  | Short arm | Long arm |
| *Cucumis sativus* | 1 | 6.79 ± 0.50 | 7.76 ± 0.19 | 14.55 ± 0.60 | 1.15 ± 0.08 | m^†^ | 1.45 ± 0.06 | 3.00 ± 0.34 | 0 | 2.90 ± 0.30 |
|  | 2 | 7.37 ± 0.21 | 9.15 ± 1.01 | 16.52 ± 1.17 | 1.24 ± 0.11 | m^†^ | 0.74 ± 0.23 | 3.93 ± 0.63 | 0 | 0 |
|  | 3 | 7.19 ± 0.83 | 7.38 ± 0.58 | 14.57 ± 1.38 | 1.03 ± 0.06 | m | 1.31 ± 0.27 | 1.74 ± 0.22 | 0 | 3.00 ± 0.33 |
|  | 4 | 5.45 ± 0.19 | 10.52 ± 0.47 | 15.97 ± 0.60 | 1.93 ± 0.07 | sm^†^ | 1.62 ± 0.07 | 3.37 ± 0.43 | 1.97 ± 0.37 | 2.27 ± 0.44 |
|  | 5 | 6.57 ± 0.38 | 7.20 ± 0.24 | 13.78 ± 0.47 | 1.10 ± 0.07 | m | 1.74 ± 0.64 | 0 | 2.23 ± 0.25 | 2.77 ± 0.48 |
|  | 6 | 5.75 ± 0.34 | 6.98 ± 0.15 | 12.73 ± 0.44 | 1.22 ± 0.06 | m | 1.60 ± 0.31 | 0 | 2.65 ± 0.27 | 0 |
|  | 7 | 5.25 ± 0.26 | 6.62 ± 0.35 | 11.88 ± 0.59 | 1.26 ± 0.03 | m | 1.41 ± 0.51 | 1.84 ± 0.25 | 1.64 ± 0.31 | 2.55 ± 0.30 |
|  | Total | 44.39 ± 0.85 | 55.61 ± 0.85 | 100 |  |  | 9.86 ± 0.48 | 13.89 ± 1.14 | 8.49 ± 0.87 | 13.49 ± 1.16 |
| *Cucumis melo* | 1 | 4.92 ± 0.71 | 5.80 ± 0.21 | 10.72 ± 0.82 | 1.19 ± 0.16 | m^†^ | 1.92 ± 0.32 | 3.59 ± 0.52 |  |  |
|  | 2 | 4.52 ± 0.35 | 5.49 ± 0.05 | 10.00 ± 0.40 | 1.22 ± 0.08 | m^†^ | 1.18 ± 0.06 | 2.42 ± 0.30 |  |  |
|  | 3 | 3.13 ± 0.20 | 5.90 ± 0.15 | 9.04 ± 0.34 | 1.89 ± 0.08 | sm | 1.86 ± 0.32 |  |  |  |
|  | 4 | 3.52 ± 0.33 | 5.39 ± 0.28 | 8.92 ± 0.36 | 1.54 ± 0.19 | m | 1.60 ± 0.34 |  |  |  |
|  | 5 | 2.25 ± 0.18 | 6.21 ± 0.07 | 8.47 ± 0.23 | 2.77 ± 0.20 | sm | 1.79 ± 0.32 |  |  |  |
|  | 6 | 3.49 ± 0.14 | 4.85 ± 0.39 | 8.33 ± 0.52 | 1.39 ± 0.07 | m | 2.04 ± 0.42 |  |  |  |
|  | 7 | 3.36 ± 0.19 | 4.64 ± 0.18 | 7.99 ± 0.19 | 1.39 ± 0.11 | m | 1.91 ± 0.11 |  |  |  |
|  | 8 | 3.28 ± 0.17 | 4.52 ± 0.08 | 7.80 ± 0.09 | 1.38 ± 0.10 | m | 1.90 ± 0.21 |  |  |  |
|  | 9 | 2.61 ± 0.22 | 4.77 ± 0.34 | 7.38 ± 0.17 | 1.84 ± 0.29 | sm | 1.97 ± 0.28 |  |  |  |
|  | 10 | 3.47 ± 0.02 | 3.88 ± 0.04 | 7.35 ± 0.04 | 1.12 ± 0.02 | m | 1.77 ± 0.05 |  |  |  |
|  | 11 | 3.26 ± 0.17 | 3.86 ± 0.12 | 7.12 ± 0.25 | 1.18 ± 0.05 | m | 1.96 ± 0.27 |  |  |  |
|  | 12 | 2.22 ± 0.15 | 4.65 ± 0.39 | 6.87 ± 0.36 | 2.11 ± 0.28 | sm | 1.73 ± 0.33 |  |  |  |
|  | Total | 40.03 ± 0.50 | 59.97 ± 0.50 | 100 |  |  | 21.62 ± 2.13 | 6.01 ± 0.54 |  |  |
| *Citrullus lanatus* | 1 | 4.83 ± 0.25 | 5.61 ± 0.18 | 10.44 ± 0.43 | 1.16 ± 0.03 | m | 3.50 ± 0.43 |  |  |  |
|  | 2 | 4.31 ± 0.20 | 5.95 ± 0.38 | 10.26 ± 0.46 | 1.38 ± 0.10 | m | 3.50 ± 0.44 |  |  |  |
|  | 3 | 3.58 ± 0.21 | 6.55 ± 0.23 | 10.14 ± 0.32 | 1.83 ± 0.13 | sm | 3.08 ± 0.63 |  |  |  |
|  | 4 | 4.02 ± 0.26 | 5.53 ± 0.42 | 9.55 ± 0.65 | 1.38 ± 0.07 | m | 2.95 ± 0.18 |  |  |  |
|  | 5 | 3.69 ± 0.18 | 5.67 ± 0.38 | 9.36 ± 0.52 | 1.54 ± 0.07 | m | 2.71 ± 0.24 |  |  |  |
|  | 6 | 3.69 ± 0.15 | 5.23 ± 0.48 | 8.92 ± 0.51 | 1.42 ± 0.14 | m | 2.99 ± 0.25 | 2.15 ± 0.19 |  |  |
|  | 7 | 4.00 ± 0.22 | 4.86 ± 0.27 | 8.85 ± 0.43 | 1.22 ± 0.06 | m | 2.82 ± 0.41 |  |  |  |
|  | 8 | 3.76 ± 0.30 | 4.56 ± 0.40 | 8.32 ± 0.31 | 1.22 ± 0.19 | m | 2.83 ± 0.29 | 2.37 ± 0.55 |  |  |
|  | 9 | 3.74 ± 0.15 | 4.54 ± 0.02 | 8.28 ± 0.15 | 1.21 ± 0.05 | m | 2.61 ± 0.47 |  |  |  |
|  | 10 | 3.21 ± 0.17 | 4.90 ± 0.25 | 8.11 ± 0.34 | 1.53 ± 0.09 | m | 2.15 ± 0.14 |  |  |  |
|  | 11 | 3.54 ± 0.26 | 4.24 ± 0.26 | 7.78 ± 0.49 | 1.20 ± 0.06 | m | 2.11 ± 0.17 |  |  |  |
|  | Total | 42.36 ± 0.54 | 57.64 ± 0.54 | 100 |  |  | 31.25 ± 1.79 | 4.52 ± 0.72 |  |  |
| *Benincasa hispida* | 1 | 5.10 ± 0.22 | 5.34 ± 0.06 | 10.44 ± 0.25 | 1.05 ± 0.04 | m |  |  |  |  |
|  | 2 | 4.68 ± 0.28 | 5.33 ± 0.11 | 10.00 ± 0.38 | 1.14 ± 0.04 | m |  |  |  |  |
|  | 3 | 3.31 ± 0.02 | 5.88 ± 0.46 | 9.19 ± 0.44 | 1.78 ± 0.15 | sm |  |  |  |  |
|  | 4 | 3.88 ± 0.19 | 5.25 ± 0.07 | 9.13 ± 0.25 | 1.35 ± 0.06 | m^†^ |  | 2.26 ± 0.17 |  |  |
|  | 5 | 4.32 ± 0.14 | 4.65 ± 0.12 | 8.97 ± 0.24 | 1.08 ± 0.03 | m |  |  |  |  |
|  | 6 | 2.57 ± 0.11 | 6.17 ± 0.24 | 8.74 ± 0.16 | 2.40 ± 0.19 | sm |  |  |  |  |
|  | 7 | 2.81 ± 0.27 | 4.76 ± 0.26 | 7.56 ± 0.02 | 1.71± 0.26 | sm |  | 1.51 ± 0.19 |  |  |
|  | 8 | 3.50 ± 0.10 | 3.97 ± 0.10 | 7.48 ± 0.02 | 1.13 ± 0.06 | m |  |  |  |  |
|  | 9 | 2.99 ± 0.13 | 4.36 ± 0.17 | 7.35 ± 0.07 | 1.46 ± 0.11 | m |  |  |  |  |
|  | 10 | 2.56 ± 0.19 | 4.73 ± 0.13 | 7.29 ± 0.10 | 1.86 ± 0.18 | sm |  |  |  |  |
|  | 11 | 3.26 ± 0.16 | 3.82 ± 0.09 | 7.07 ± 0.24 | 1.17 ± 0.04 | m |  |  |  |  |
|  | 12 | 2.61 ± 0.14 | 4.17 ± 0.18 | 6.78 ± 0.29 | 1.60 ± 0.06 | m |  |  |  |  |
|  | total | 41.58 ± 0.69 | 58.42 ± 0.69 | 100 |  |  |  | 3.77 ± 0.36 |  |  |
| *Momordica charantia* | 1 | 5.44 ± 0.66 | 6.19 ± 0.36 | 11.63 ± 1.01 | 1.14± 0.07 | m | 2.48 ± 0.11 |  |  |  |
|  | 2 | 4.52 ± 0.74 | 6.53 ± 0.33 | 11.05 ± 1.06 | 1.47 ± 0.19 | m^†^ | 1.87 ± 0.37 | 3.28 ± 0.26 |  |  |
|  | 3 | 4.83 ± 0.16 | 5.26 ± 0.39 | 10.09 ± 0.50 | 1.09 ± 0.07 | m | 2.56 ± 0.42 |  |  |  |
|  | 4 | 4.01 ± 0.84 | 5.94 ± 0.41 | 9.94 ± 1.10 | 1.52 ± 0.26 | m^†^ | 1.67 ± 0.23 | 3.67 ± 1.11 |  |  |
|  | 5 | 4.46 ± 0.05 | 5.16 ± 0.20 | 9.61 ± 0.19 | 1.16 ± 0.05 | m | 2.28 ± 0.27 |  |  |  |
|  | 6 | 3.95 ± 0.35 | 4.92 ± 0.31 | 8.87 ± 0.55 | 1.25 ± 0.11 | m | 2.22 ± 0.35 |  |  |  |
|  | 7 | 3.33 ± 0.36 | 5.15 ± 0.16 | 8.48 ± 0.51 | 1.56 ± 0.13 | m | 2.20 ± 0.53 |  |  |  |
|  | 8 | 3.01 ± 0.33 | 4.92 ± 0.53 | 7.93 ± 0.86 | 1.63 ± 0.01 | m | 1.85 ± 0.26 |  |  |  |
|  | 9 | 2.60 ± 0.01 | 5.31 ± 0.20 | 7.91 ± 0.20 | 2.04 ± 0.08 | sm | 2.00 ± 0.42 |  |  |  |
|  | 10 | 3.57 ± 0.14 | 4.29 ± 0.19 | 7.86 ± 0.32 | 1.20 ± 0.02 | m | 1.80 ± 0.33 |  |  |  |
|  | 11 | 2.98 ± 0.34 | 3.65 ± 0.22 | 6.64 ± 0.15 | 1.24 ± 0.22 | m | 2.00 ± 0.18 |  |  |  |
|  | total | 42.69 ± 0.87 | 57.31± 0.87 | 100.00 |  |  | 22.92 ± 2.97 | 6.95 ± 0.96 |  |  |
| *Luffa cylindrica* | 1 | 4.74 ± 0.54 | 5.67 ± 0.19 | 10.41 ± 0.74 | 1.21 ± 0.10 | m | 1.99 ± 0.11 | 1.83 ± 0.50 |  |  |
|  | 2 | 4.44 ± 0.51 | 5.55 ± 0.10 | 9.99 ± 0.57 | 1.26 ± 0.14 | m | 2.21 ± 0.42 | 1.28 ± 0.22 |  |  |
|  | 3 | 4.29 ± 0.31 | 5.27 ± 0.15 | 9.56 ± 0.45 | 1.23 ± 0.06 | m | 1.86 ± 0.31 |  |  |  |
|  | 4 | 4.24 ± 0.15 | 5.17 ± 0.34 | 9.41 ± 0.32 | 1.22 ± 0.11 | m | 2.15 ± 0.39 |  |  |  |
|  | 5 | 4.03 ± 0.29 | 5.09 ± 0.37 | 9.12 ± 0.63 | 1.26 ± 0.05 | m | 1.80 ± 0.29 | 1.22 ± 0.29 |  |  |
|  | 6 | 4.36 ± 0.19 | 4.73 ± 0.18 | 9.09 ± 0.37 | 1.08 ± 0.02 | m | 2.18 ± 0.29 |  |  |  |
|  | 7 | 3.44 ± 0.53 | 5.27 ± 0.26 | 8.71 ± 0.79 | 1.55 ± 0.16 | m | 2.12 ± 0.19 |  |  |  |
|  | 8 | 4.22 ± 0.36 | 4.34 ± 0.37 | 8.56 ± 0.70 | 1.03 ± 0.05 | m | 1.99 ± 0.29 | 1.22 ± 0.15 |  |  |
|  | 9 | 4.08 ± 0.26 | 4.48 ± 0.13 | 8.56 ± 0.29 | 1.10 ± 0.08 | m | 2.02 ± 0.33 |  |  |  |
|  | 10 | 4.05 ± 0.35 | 4.50 ± 0.34 | 8.55 ± 0.68 | 1.11 ± 0.03 | m | 2.15 ± 0.15 |  |  |  |
|  | 11 | 3.81 ± 0.14 | 4.26 ± 0.25 | 8.07 ± 0.34 | 1.12 ± 0.06 | m | 1.99 ± 0.20 |  |  |  |
|  | 12 | 3.46 ± 0.54 | 4.58 ± 0.46 | 8.05 ± 0.89 | 1.34 ± 0.19 | m | 1.73 ± 0.19 | 1.03 ± 0.11 |  |  |
|  | 13 | 3.26 ± 0.29 | 3.77 ± 0.46 | 7.03 ± 0.69 | 1.16 ± 0.10 | m | 1.70 ± 0.20 |  |  |  |
|  | Total | 45.34 ± 0.15 | 54.66 ± 0.15 | 100.00 |  |  | 25.89 ± 1.93 | 6.58 ± 0.81 |  |  |
| *Lagenaria siceraria* var. *hispida* | 1 | 5.78 ± 0.26 | 8.73 ± 0.27 | 14.52 ± 0.51 | 1.51 ± 0.04 | m^†^ |  | 4.89 ± 0.30 |  |  |
|  | 2 | 4.90 ± 0.19 | 6.40 ± 0.24 | 11.30 ± 0.38 | 1.31 ± 0.05 | m |  |  |  |  |
|  | 3 | 4.76 ± 0.31 | 5.30 ± 0.34 | 10.06 ± 0.41 | 1.12 ± 0.12 | m |  |  |  |  |
|  | 4 | 4.00 ± 0.23 | 5.45 ± 0.13 | 9.45 ± 0.10 | 1.37 ± 0.11 | m |  |  |  |  |
|  | 5 | 4.02 ± 0.42 | 4.83 ± 0.24 | 8.85 ± 0.18 | 1.21 ± 0.20 | m |  |  |  |  |
|  | 6 | 2.95 ± 0.04 | 5.55 ± 0.35 | 8.50 ± 0.39 | 1.88 ± 0.10 | sm |  | 1.89 ± 0.3 |  |  |
|  | 7 | 3.50 ± 0.34 | 4.62 ± 0.20 | 8.11 ± 0.40 | 1.33 ± 0.13 | m |  |  |  |  |
|  | 8 | 3.35 ± 0.35 | 4.69 ± 0.21 | 8.04 ± 0.26 | 1.41 ± 0.19 | m |  |  |  |  |
|  | 9 | 3.26 ± 0.59 | 4.19 ± 0.27 | 7.45 ± 0.74 | 1.31 ± 0.22 | m |  |  |  |  |
|  | 10 | 2.81 ± 0.15 | 4.24 ± 0.57 | 7.05 ± 0.59 | 1.51 ± 0.23 | m |  |  |  |  |
|  | 11 | 2.67 ± 0.11 | 4.00 ± 0.49 | 6.67 ± 0.58 | 1.50 ± 0.14 | m |  |  |  |  |
|  | Total | 41.99 ± 0.96 | 58.01 ± 0.96 | 100.00 |  |  |  | 6.77 ± 0.30 |  |  |
| *Cucurbita moschata* |  |  |  |  |  |  |  |  | Pericentromeric DAPI^+^ band size^‡^ ±SD |  |
|  | 1 | 2.79 ± 0.18 | 3.83 ± 0.18 | 6.63 ± 0.35 | 1.37 ± 0.03 | m |  |  |  |  |
|  | 2 | 2.92 ± 0.21 | 3.70 ± 0.25 | 6.61 ± 0.11 | 1.26 ± 0.16 | m |  | 1.99 ± 0.21 |  |  |
|  | 3 | 2.18 ± 0.20 | 4.23 ± 0.14 | 6.41 ± 0.32 | 2.00 ± 0.07 | sm |  | 1.79 ±0.36 |  |  |
|  | 4 | 2.51 ± 0.24 | 3.25 ± 0.24 | 5.76 ± 0.39 | 1.29 ± 0.13 | m |  | 1.32 ±0.19 |  |  |
|  | 5 | 2.29 ± 0.10 | 3.08 ± 0.19 | 5.37 ± 0.19 | 1.35 ± 0.12 | m |  |  |  |  |
|  | 6 | 2.24 ± 0.20 | 3.11 ± 0.38 | 5.35 ± 0.43 | 1.46 ± 0.15 | m |  | 0.83 ±0.12 |  |  |
|  | 7 | 2.07 ± 0.18 | 3.16 ± 0.44 | 5.23 ± 0.60 | 1.48 ± 0.08 | m |  |  | 0.97 ± 0.14 |  |
|  | 8 | 2.28 ± 0.36 | 2.93 ± 0.13 | 5.20 ± 0.49 | 1.34 ± 0.11 | m |  |  |  |  |
|  | 9 | 2.28 ± 0.28 | 2.80 ± 0.10 | 5.07 ± 0.36 | 1.21 ± 0.10 | m |  |  |  |  |
|  | 10 | 2.31 ± 0.20 | 2.74 ± 0.29 | 5.05 ± 0.42 | 1.15 ± 0.08 | m |  |  | 0.83 ± 0.01 |  |
|  | 11 | 2.18 ± 0.02 | 2.77 ± 0.35 | 4.95 ± 0.33 | 1.22 ± 0.12 | m |  |  | 1.96 ± 0.20 |  |
|  | 12 | 2.17 ± 0.18 | 2.71 ± 0.20 | 4.88 ± 0.33 | 1.23 ± 0.08 | m |  | 0.68 ±0.09 |  |  |
|  | 13 | 2.02 ± 0.14 | 2.68 ± 0.20 | 4.70 ± 0.08 | 1.31 ± 0.17 | m |  |  |  |  |
|  | 14 | 2.16 ± 0.12 | 2.31 ± 0.16 | 4.47 ± 0.27 | 1.06 ± 0.01 | m |  |  |  |  |
|  | 15 | 1.94 ± 0.18 | 2.39 ± 0.22 | 4.33 ± 0.29 | 1.23 ± 0.16 | m |  |  |  |  |
|  | 16 | 1.97 ± 0.06 | 2.27 ± 0.04 | 4.24 ± 0.10 | 1.14 ± 0.01 | m |  |  |  |  |
|  | 17 | 1.86 ± 0.29 | 2.29 ± 0.33 | 4.15 ± 0.50 | 1.30 ± 0.19 | m |  |  |  |  |
|  | 18 | 1.95 ± 0.21 | 2.20 ± 0.11 | 4.14 ± 0.29 | 1.09 ± 0.03 | m |  |  | 1.13 ± 0.08 |  |
|  | 19 | 1.77 ± 0.30 | 2.28 ± 0.54 | 4.05 ± 0.84 | 1.32 ± 0.03 | m |  |  |  |  |
|  | 20 | 1.63 ± 0.18 | 1.78 ± 0.07 | 3.40 ± 0.24 | 1.13 ± 0.05 | m |  |  |  |  |
|  | Total | 43.49 ± 0.16 | 56.51 ± 0.16 | 100 |  |  |  | 6.60 ± 0.26 | 4.88 ± 0.17 |  |

SD, standard deviation. m, metacentric. ^†^satellite chromosome (satellite length was included in chromosome length but secondary constriction length was excluded). ^‡^ % of band size in relation to the karyotype length.
