# Supplementary material for: ﻿Comparative karyotype analysis of eight Cucurbitaceae crops using fluorochrome banding and 45S rDNA-FISH
Source: Comp Cytogenet. 2023 Feb 9;17:31–58. doi: 10.3897/compcytogen.17.99236 (PMC10252140; doi:10.3897/compcytogen.17.99236)
Supplement: Supplementary material 4 — The correlational analysis between the difference in TCL and the change in nuclear DNA content within the eight Cucurbitaceae crops using the SPSS 25.0 software [file comparative_cytogenetics-17--031_article-99236__-s004.docx]

**Table S3. The correlational analysis between the difference in TCL and the change in nuclear DNA content within the eight Cucurbitaceae crops using the SPSS 25.0 software.**

|  |  | Nuclear DNA content | TCL |
| --- | --- | --- | --- |
| Nuclear DNA content | Pearson product-moment correlation coefficient | 1 | 0.899 |
|  | Sig. (2-tailed) |  | 0.002 |
|  | Cases | 8 | 8 |
| TCL | Pearson product-moment correlation coefficient | 0.899 | 1 |
|  | Sig. (2-tailed) | 0.002 |  |
|  | Cases | 8 | 8 |
