# Supplementary material for: ﻿Comparative karyotype analysis of eight Cucurbitaceae crops using fluorochrome banding and 45S rDNA-FISH
Source: Comp Cytogenet. 2023 Feb 9;17:31–58. doi: 10.3897/compcytogen.17.99236 (PMC10252140; doi:10.3897/compcytogen.17.99236)
Supplement: Supplementary material 5 — The number and position of 45S rDNA locus in Cucurbitaceae species [file comparative_cytogenetics-17--031_article-99236__-s005.docx]

**Table S4. The number and position of 45S rDNA locus in Cucurbitaceae species**

| Species | Number of chromosome | Number of 45S rDNA locus | Position of 45S rDNA | Reference |
| --- | --- | --- | --- | --- |
| *Acanthosicyos naudinianus*^＊^ | 2n=24 | 2 | All TER | Li et al. 2016 |
| *Actinostemma tenerum* | 2n=16 | 1 | PROX | Pellerin et al. 2018b |
| *Benincasa fistulosa*^＊^ | 2n=24 | 2 | All TER | Li et al. 2016 |
| *Benincasa hispida*^＊^ | 2n=24 | 2 | All TER | This study; Xie et al 2019 |
| *Citrullus colocynthis*^＊^ | 2n=22 | 2 | All TER | Li et al. 2016 |
| *Citrullus ecirrhosus*^＊^ | 2n=22 | 1 | TER | Li et al. 2016 |
| *Citrullus lanatus*^＊^ | 2n=22 | 2 | All TER | This study; Xie et al 2019 |
| *Citrullus lanatus* subsp. *lanatus*^＊^ | 2n=22 | 1 | TER | Li et al. 2016 |
| *Citrullus lanatus* subsp. *mucosospermus*^＊^ | 2n=22 | 2 | All TER | Li et al. 2016 |
| *Citrullus lanatus* subsp. *vulgaris*^＊^ | 2n=22 | 2 | All TER | Li et al. 2016 |
| *Citrullus lanatus var. citroides*^＊^ | 2n=22 | 1 | TER | Waminal & Kim 2015 |
| *Citrullus rehmii*^＊^ | 2n=22 | 1 | TER | Li et al. 2016 |
| *Coccinia grandis*^＊^ | 2n=24 | 2 | All TER | Xie et al. 2019 |
| *Coccinia sessilifolia*^＊^ | 2n=24 | 3 | All TER | Li et al. 2016 |
| *Cucumis africanus*^＊^ | 2n=24 | 3 | Two TER, one PROX | Yagi et al. 2015；Zhang et al. 2016 |
| *Cucumis anguria*^＊^ | 2n=24 | 2 | All TER | Zhang et al. 2015a；Zhang et al. 2016 |
| *Cucumis asper*^＊^ | 2n=24 | 2 | All TER | Zhang et al. 2016 |
| *Cucumis dipsaceus*^＊^ | 2n=24 | 2 | All TER | Zhang et al. 2016 |
| *Cucumis ficifolius*^＊^ | 2n=48 | 2 | All TER | Zhang et al. 2016 |
| *Cucumis heptadactylus*^＊^ | 2n=48 | 6 | Four TER, two PROX | Yagi et al. 2015 |
| *Cucumis heptadactylus*^＊^ | 2n=48 | 4 | All TER | Zhang et al. 2016 |
| *Cucumis hystrix*^＊^ | 2n=24 | 3 | All TER | Zhang et al. 2015a； Zhang et al. 2016 |
| *Cucumis meeusei*^＊^ | 2n=48 | 4 | three TER, one PROX | Zhang et al. 2016 |
| *Cucumis melo*^＊^ | 2n=24 | 2 | One TER, one PROX | This study; Xie et al 2019 |
| *Cucumis melo var. flexuosus*^＊^ | 2n=24 | 2 | One TER, one PROX | Pellerin et al. 2018 |
| *Cucumis metuliferus*^＊^ | 2n=24 | 2 | All TER | Zhang et al. 2015a； Zhang et al. 2016； Li et al. 2016 |
| *Cucumis myriocarpus*^＊^ | 2n=24 | 3 | Two TER, one PROX | Yagi et al. 2015 |
| *Cucumis myriocarpus*^＊^ | 2n=24 | 2 | All TER | Zhang et al. 2016 |
| *Cucumis pustulatus*^＊^ | 2n=72 | 5 | All TER | Zhang et al. 2016 |
| *Cucumis sativus*^＊^ | 2n=14 | 5 | All PROX | This study |
| *Cucumis sativus var. hardwickii*^＊^ | 2n=14 | 3 | All PROX | Zhao et al. 2011 |
| *Cucumis sativus var. sativus*^＊^ | 2n=14 | 5 | All PROX | Zhao et al. 2011 |
| *Cucumis sativus var. xishuangbannesis*^＊^ | 2n=14 | 5 | All PROX | Zhao et al. 2011 |
| *Cucumis subsericeus*^＊^ | 2n=48 | 1 | TER | Zhang et al. 2016 |
| *Cucumis zambianus*^＊^ | 2n=24 | 2 | All TER | Zhang et al. 2016 |
| *Cucumis zeyheri*^＊^ | 2n=24 | 2 | All TER | Yagi et al. 2015 |
| *Cucurbita moschata* | 2n=40 | 5 | All PROX | This study |
| *Cucurbita moschata* | 2n=40 | 4 | All PROX | Xie et al 2019 |
| *Cucurbita pepo* | 2n=40 | 5 | Four PROX, one PCEN | Xie et al 2019 |
| *Diplocyclos palmatus*^＊^ | 2n=24 | 3 | All TER | Li et al. 2016 |
| *Gymnopetalum chinense* | 2n=22 | 3 | All TER | Xie et al 2019 |
| *Gynostemma pentaphyllum* | 2n=66 | 5 | All TER | Pellerin et al. 2018b |
| *Herpetospermum pedunculosum* | 2n=20 | 7 | One TER, three INTER, three PROX | Xie et al 2019 |
| *Lagenaria siceraria*^＊^ | 2n=22 | 2 | All TER | Li et al. 2016; Xie et al 2019 |
| *Lagenaria siceraria* var. *hispida*^＊^ | 2n=22 | 2 | All TER | This study |
| *Luffa cylindrica* | 2n=26 | 5 | All TER | This study |
| *Luffa cylindrica* | 2n=26 | 2 | All TER | Xie et al 2019 |
| *Melothria japonica*^＊^ | 2n=22 | 2 | All PROX | Waminal & Kim 2015 |
| *Melothria pendula*^＊^ | 2n=24 | 2 | One TER, one PROX | Li et al. 2016 |
| *Melothria pendula*^＊^ | 2n=24 | 4 | One TER, thee PROX | Pellerin et al. 2018 |
| *Momordica charantia* | 2n=22 | 2 | All TER | This study; Xie et al 2019 |
| *Momordica cochinchinensis* | 2n=28 | 4 | All TER | Xie et al 2019 |
| *Momordica dioica* | 2n=24 | 2 | All TER | Xie et al 2019 |
| *Sechium edule* | 2n=28 | 3 | Two PROX, one TER | Pellerin et al. 2018b |
| *Sechium edule* | 2n=28 | 2 | One TER, one PROX | Xie et al 2019 |
| *Sicyos angulatus* | 2n 24 | 2 | All PROX | Waminal & Kim 2015 |
| *Siraitia grosvenorii* | 2n=28 | 3 | All TER | Xie et al 2019; Li et al. 2007 |
| *Thladiantha dubia* | 2n=18 | 3 | All TER | Pellerin et al. 2018b |
| *Trichosanthes anguina* | 2n=22 | 3 | All TER | Pellerin et al. 2018; Xie et al 2019 |
| *Trichosanthes dunniana* | 2n=20 | 3 | All TER | Xie et al 2019 |
| *Trichosanthes kirilowii* | 2n = 66, 88, 110 | 5 | All TER | Waminal & Kim 2015 |
| *Trichosanthes ovigera* | 2n=22 | 5 | All TER | Xie et al 2019 |
| *Zehneria mariothii*^＊^ | 2n=24 | 2 | One TER, one PROX | Li et al. 2016 |
| *Zehneria maysorensis*^＊^ | 2n=24 | 1 | 1TER | Xie et al 2019 |

^＊^Indicate the species belonging to tribe Benincaseae (Kocyan et al. 2007).
